# Groucho related gene 5 (GRG5) is involved in embryonic and neural stem cell state decisions

**DOI:** 10.1038/s41598-018-31696-9

**Published:** 2018-09-13

**Authors:** Konstantina Chanoumidou, Christiana Hadjimichael, Paraskevi Athanasouli, Henrik Ahlenius, Antonis Klonizakis, Christoforos Nikolaou, Elias Drakos, Antonis Kostouros, Irene Stratidaki, Maria Grigoriou, Androniki Kretsovali

**Affiliations:** 1 Department of Molecular Biology and Genetics, Democritus University of Thrace, 68100 Alexandroupoli, Greece; 2 Institute of Molecular Biology and Biotechnology, Foundation for Research and Technology-Hellas (FORTH), 70013 Heraklion, Crete Greece; 3 Lund Stem Cell Center, University Hospital, SE-221 84 Lund, Sweden; 4 Department of Biology, University of Crete, 71409 Heraklion, Crete Greece; 5 School of Medicine, University of Crete, 71003 Heraklion, Crete Greece

## Abstract

Groucho related gene 5 (GRG5) is a multifunctional protein that has been implicated in late embryonic and postnatal mouse development. Here, we describe a previously unknown role of GRG5 in early developmental stages by analyzing its function in stem cell fate decisions. By both loss and gain of function approaches we demonstrate that ablation of GRG5 deregulates the Embryonic Stem Cell (ESC) pluripotent state whereas its overexpression leads to enhanced self-renewal and acquisition of cancer cell-like properties. The malignant characteristics of teratomas generated by ESCs that overexpress GRG5 reveal its pro-oncogenic potential. Furthermore, transcriptomic analysis and cell differentiation approaches underline GRG5 as a multifaceted signaling regulator that represses mesendodermal-related genes. When ESCs exit pluripotency, GRG5 promotes neuroectodermal specification via Wnt and BMP signaling suppression. Moreover, GRG5 promotes the neuronal reprogramming of fibroblasts and maintains the self-renewal of Neural Stem Cells (NSCs) by sustaining the activity of Notch/Hes and Stat3 signaling pathways. In summary, our results demonstrate that GRG5 has pleiotropic roles in stem cell biology functioning as a stemness factor and a neural fate specifier.

## Introduction

Embryonic stem cells (ESCs) are characterized by self-renewal and pluripotency, properties that enable large-scale generation of any somatic cell type. The equilibrium between pluripotency and differentiation is regulated by a complex network centered around the triad of the OCT4, SOX2 and NANOG transcription factors^1,2^. Moreover, signaling pathways that respond to the extracellular milieu play equally important roles. For murine ESCs LIF/Jak/Stat3, Wnt and Bmp signaling cascades are considered critical regulators of both self-renewal and cell fate decision^3–7^.

A wealth of recent studies has focused on ESC neural differentiation to study the development of central nervous system during embryogenesis and its disorders due to shared molecular mechanisms^8^. In this regard, the establishment of neuroectoderm is considered as default fate upon suppression of the mesendoderm promoting signals Wnt, Bmp and Activin/Nodal^9–12^. Recently, the accomplishment of direct neuronal reprogramming of somatic cells^13–16^ has provided an additional valuable system to identify neural fate determinants and understand the regeneration of neuronal tissue.

The Groucho/TLE/GRG family members are versatile transcriptional co-factors with important role in multiple developmental processes through regulation of Notch, Wnt and RTK pathways^17–20^. Well established is their conserved role in neurogenesis regulation, where they act as co-repressors of critical transcription factors including HES1 and FOXG1^21–23^. Moreover, they have emerged as direct or indirect effectors of various neoplasias including leukemias, brain, hepatic and pancreatic cancers^24,25^. In mammals, the Groucho related gene (GRG) family is subdivided in two protein groups that present different size and antagonistic function, the long GRGs (GRG 1–4) and the truncated family members (GRG5, 6). GRG5 (the mouse ortholog of human AES) is a multifunctional protein implicated in different cellular processes including transcriptional regulation, apoptosis and cancer development via interaction with critical signaling mediators^26^. Over the past decade, studies have characterized AES as tumor suppressor^27–29^, however its oncogenic property has been reported in AML^30,31^. GRG5 has active role in various developmental processes of the late embryonic and postnatal period with most important its function in osteogenesis, where it regulates RUNX2 activity^32–34^. However, its function in early developmental stages has not been explored yet.

GRG5 is the Groucho member that shows the highest expression in undifferentiated ESCs and becomes down-regulated upon differentiation^35,36^. Although GRG5 has been reported as a direct transcriptional target of STAT3 in ESCs^37^, whether it is involved in pluripotent cell maintenance and/or specification remains unknown.

In this study, we investigate for the first time the function of GRG5 in mouse ESCs and embryonic NSCs. We show that ablation of GRG5 deregulates ESC pluripotency, whereas its overexpression leads to enhanced self-renewal and acquisition of cancer cell-like properties. Moreover, we reveal the neurogenic potential of GRG5 by demonstrating that it is required for the neuroectodermal specification of ESCs, neuronal reprogramming of fibroblasts and maintenance of embryonic NSC identity.

## Results

### Loss of GRG5 deregulates ESC pluripotent state

**Figure 1:**
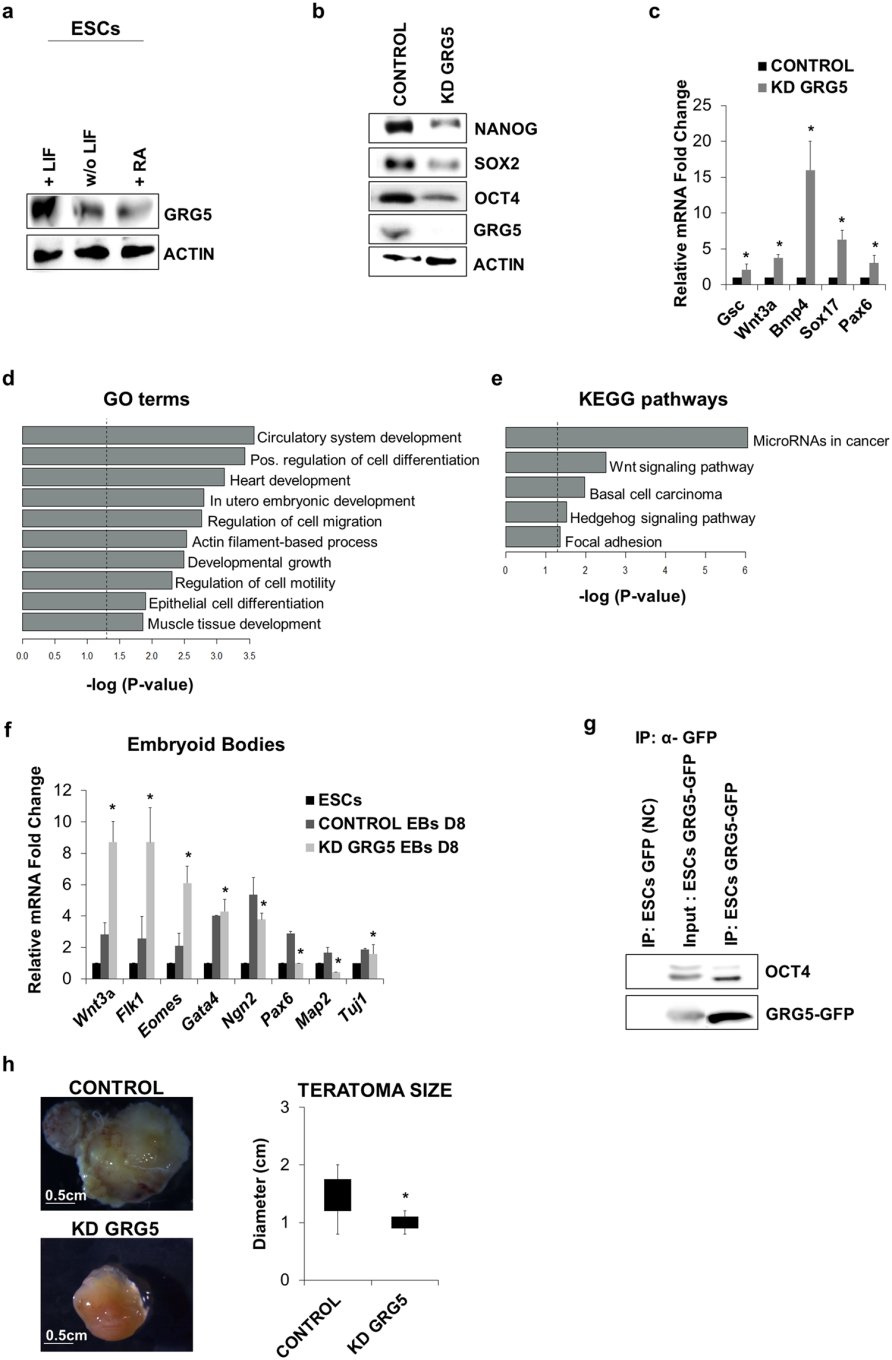
GRG5 is highly expressed in ESCs and its knock-down destabilizes cell pluripotent state. (**a**) Western Blot analysis of GRG5 in ESCs cultured in pluripotency promoting conditions (+LIF) and upon induction of differentiation through LIF withdrawal (w/o LIF) and 1 μM Retinoic Acid treatment (RA) for 3 days. (**b**) Protein levels of pluripotency factors upon depletion of GRG5. (**c**) Relative mRNA levels of differentiation markers in CONTROL and KD GRG5 ESCs. Mean + SD of n = 3 independent experiments. *P < 0.05 (**d**) Genes over-expressed in KD GRG5 ESCs were tested using g:Profiler^73^. The presented gene ontology (GO) terms belong to the top 20 of the significantly enriched GOs (log2FC ≥ 0.65, p-value ≤ 0.05, up to 1000 genes per GO). (**e**) Bar graph showing significantly enriched KEGG pathways. KEGG enrichment analysis was performed for the over-expressed genes in KD GRG5 ESCs using g:Profiler. (log2FC ≥ 0.65, p-value ≤ 0.05) (**f**) Differentiation of CONTROL and KD GRG5 ESCs through EBs formation. Mesodermal (*Wnt3a, Flk1, Eomes*), endodermal (*Gata4*) and neuroectodermal (*Ngn2, Pax6, Map2, Tuj1*) gene expression levels at Day8 of cell differentiation. Mean + SD of N = 4 independent experiments. *P < 0.05 (**g**) Co-immunoprecipitation assay showing interaction of GRG5 with OCT4 in ESCs. Whole cell lysates of ESCs expressing GFP (Negative control) or GRG5-GFP were immunoprecipitated with anti-GFP and immunoblotted with anti-OCT4. (**h**) Teratoma formation upon intramuscular injection of CONTROL and KD GRG5 ESCs in immunocompromised mice. Photos of the generated teratomas. (Scale bar, 0.5 cm). Box plot analysis of CONTROL and KD GRG5 teratoma size. Mean ± SD of n = 4 independent teratomas. *P < 0.05.

To examine whether GRG5 is involved in mouse ESC function, we first analyzed its expression prior and upon induction of cell differentiation through leukemia inhibitory factor (LIF) withdrawal or Retinoic Acid (RA) treatment. Western blot analysis showed GRG5 to be highly expressed in undifferentiated cells, whereas its expression declines upon cell exit from pluripotency (Fig. 1a).

We then performed knockdown (KD) of GRG5 with lentivirus expressing specific short hairpin RNA (shRNA). Two KD ESC lines were generated using independent shRNAs to exclude off-target effects and pools of the infected cells were selected based on antibiotic resistance. Both cell lines presented similar behavior. The most representative cell line (KD GRG5) is shown in Fig. 1 while the analysis of the second cell line (shRNA 17) is presented in the Supplementary Fig. S1. No significant difference in colony morphology was observed (data not shown). GRG5 depletion led to decreased protein levels of the core pluripotency factors (Fig. 1b, Supplementary Fig. S1a) and concomitant transcriptional de-repression of developmental markers of all three germ layers (Fig. 1c, Supplementary Fig. S1b). In an effort to unravel the molecular mechanisms in which GRG5 operates in ESCs we performed RNA-seq followed by validation with q-PCR for selected genes (Supplementary Fig. S1c). Out of 430 genes that were differentially expressed in the absence of GRG5, 310 were overexpressed whereas 120 were under-expressed indicating that in ESCs GRG5 acts predominantly as a co-repressor (Table S1, log2FC ≥ 0.65, p-value ≤ 0.05, Supplementary Excel). Notably, the mRNA levels of the pluripotency factors were found unaffected suggesting that their decreased protein levels resulted from changes at the post-transcriptional level (Supplementary Fig. S1d).

Gene ontology (GO) enrichment analysis (Table S2, Supplementary Excel) of genome-wide expression profiles upon GRG5 KD highlighted cell differentiation processes as being significantly over-represented in KD GRG5 ESCs (Fig. 1d). Strikingly, the majority of the upregulated genes are implicated in mesendodermal differentiation (circulatory system, heart and muscle tissue development) indicating a differentiation bias (Fig. 1d, Table 1). In accordance, KEGG pathway analysis of the overexpressed genes showed that Wnt signaling pathway, known to be important for primitive streak development, was significantly enriched (Fig. 1e, Table 1)^38,39^. Within the up-regulated genes we also identified several components of the Bmp signaling pathway that has been implicated in cardiovascular commitment of pluripotent cells^40^ (Table 1). In addition, we noticed the altered expression of many imprinted genes in the absence of GRG5.

**Table 1:** Representative differentially expressed genes related to ESC differentiation state, Wnt and Bmp signaling pathway and genomic imprinting upon GRG5 knockdown in ESCs.

Gene	log2FC	p-value
**ESC differentiation**		
OTX2	0.643397811150491	0.0217809032420395
HAND1	0.838197930573174	0.00582682146607644
GATA3	0.657157616377971	0.00684470490081028
PAX6	0.665198998525084	0.031801996764787
IGF2	1.94751094016799	1.86830422029062e-11
FGFR2	0.886441914782073	0.00418421017283026
TLE3	0.597942035513093	0.00828458468586543
TLE4	0.685573860808661	0.0111701351633792
EPRN	1.736573202	0.00000000668
**Muscle tissue**		
ERBB3	1.17140474765219	3.12801610930891e-05
ACTA1	0.81865658760647	0.00279903589867089
TTN	1.007658276159	0.000341209182525252
SRF	0.793358218843073	0.00505349410666154
FLNB	0.69022670234959	0.00187508010018393
ARID1A	0.756912548056043	0.00398935257473487
FHL2	0.893234316946676	0.0011651343898939
DSP	0.778191337241334	0.00199626027442512
SMYD1	1.45094015682622	2.28451381780708e-06
MYOF	0.952954637050711	3.09369774133293e-05
**Wnt signaling**		
WNT7B	0.650652715932638	0.0110766467218801
TCF7L1	0.867909681166531	0.0021533960601604
TCF7L2	0.828192954007797	0.00754557719898023
PORCN	0.787978629924923	0.0013905149372164
GPC4	0.713668305849584	0.0192614128047571
CREBBP	0.741726904869622	0.00822987020893197
CCND1	0.69067643023343	0.00846016520291196
CCND2	0.927654802529013	0.00266061239292888
INVS	0.790740515486736	0.0105647418864285
VANGL1	0.709423216915585	0.00133857844662215
**Bmp signaling**		
BMP8a	0.747235401967705	0.0134073677051266
BMP1	0.688064855826583	0.0245331165752351
SMAD6	0.844965702988924	0.00418936224770328
SMAD7	0.606677329643133	0.0221729087437108
ID2	0.550892365399361	0.0489249485706468
GDF3	0.574826833157946	0.0262475890669595
GATA3	0.657157616377971	0.00684470490081028
**Genomic imprinting**		
RIAN	−3.3823585382425	2.23191272070603e-30
MEG3	−3.16335509038221	4.31192431937524e-27
H19	−1.43302541629794	3.18774206765781e-06
PEG10	1.12895482676553	3.25687156497012e-05
RHOX5	1.93229192989308	2.28564655038052e-17

We then assessed the impact of GRG5 depletion in ESC differentiation potential through Embryoid bodies (EBs) formation. In line with the RNA-seq results, qPCR analysis of representative differentiation factors revealed that the KD GRG5 EBs are characterized by higher induction of mesendodermal genes (*Wnt3a, Flk1, Eomes, Gata4*). In contrast, they express lower amounts of neuroectodermal markers (*Ngn2, Pax6, Map2, Tuj1*) indicating that GRG5 influences ESC differentiation decisions in favor of neuroectoderm (Fig. 1f, Supplementary Fig. S1e).

Considering the transcriptomic changes and the co-factor nature of GRG5 we searched for putative interaction with the core pluripotency factors. Co-immunoprecipitation assays revealed GRG5 physical association with the master transcriptional regulator OCT4 (Fig. 1g), but not with Nanog (data not shown). Importantly, comparison of our data with genome wide expression analysis of KD OCT4 ESCs showed GRG5 targets to be significantly overlapping with the targets of OCT4 (Supplementary Fig. S2), highlighting a functional relevance to their interaction. These findings place GRG5 into the OCT4 complex interaction network and suggest that it performs a regulatory function in ESCs.

Finally, in an attempt to further delineate the function of GRG5 in ESCs, we tested the ability of KD GRG5 ESCs to form teratomas. Cell injection into immunocompromised mice showed that KD GRG5 ESCs give rise to teratomas of markedly smaller size as compared to the CONTROL hinting reduced self-renewal capacity (Fig. 1h). Taken together, out results indicate that GRG5 contributes to the maintenance of ESC pluripotency.

### GRG5 overexpression enhances ESC self-renewal and their tumorigenic potential

**Figure 2:**
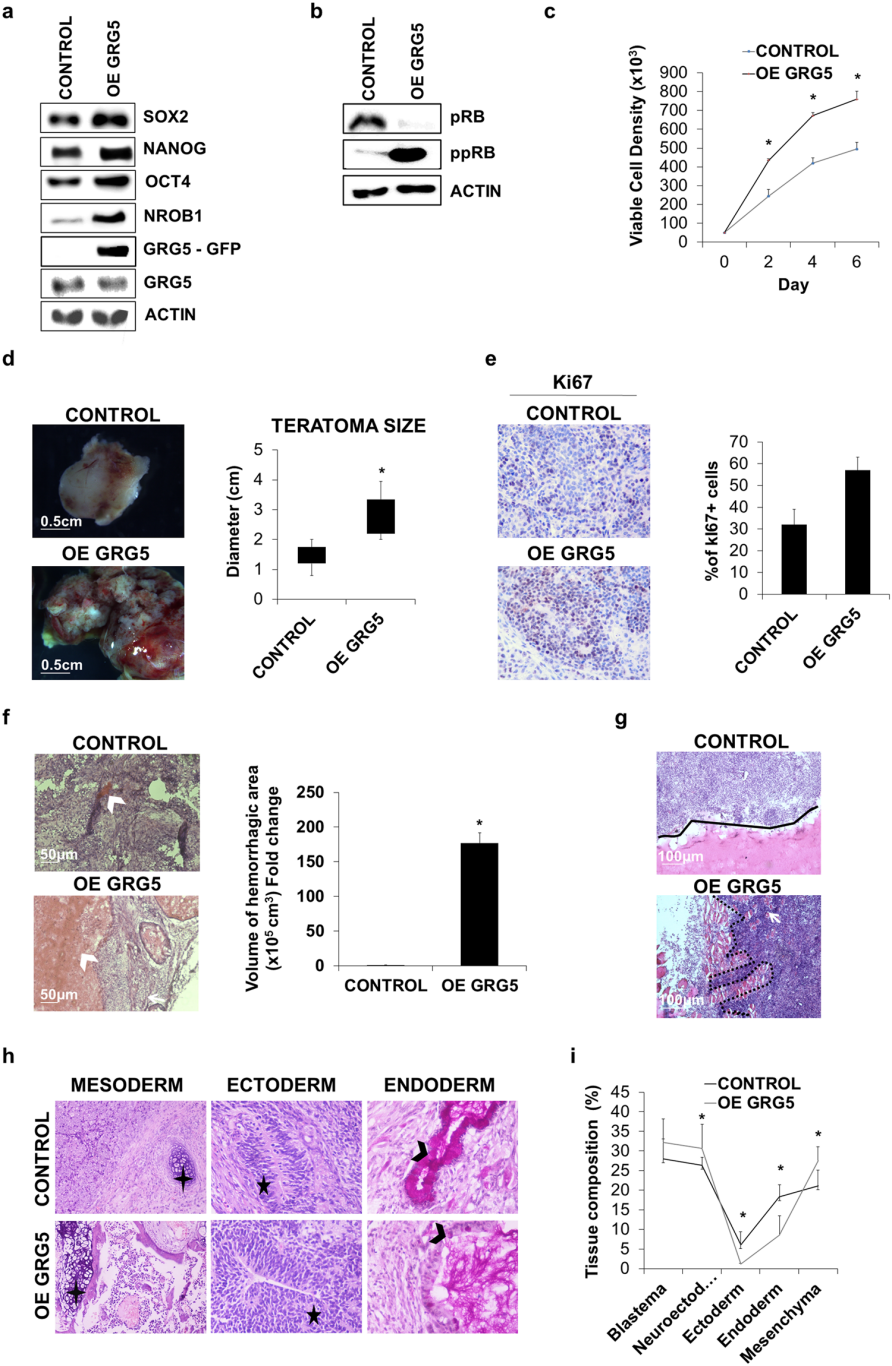
GRG5 over-expression enhances ESC self-renewal and leads to the generation of malignant teratomas. (**a**) Western Blot analysis of pluripotency factors in CONTROL and OE GRG5 ESCs. (**b**) Protein levels of total (pRB) and inactive (ppRB) RB protein in CONTROL and OE GRG5 ESCs. (**c**) Growth curves of CONTROL and OE GRG5 ESCs. Mean ± SD of n = 4 independent experiments. *P < 0.05 (**d**) Teratomas formed upon intramuscular injection of CONTROL and OE GRG5 ESCs in immunocompromised mice. (Scale bar, 0.5 cm) Box plot illustrating the average size of CONTROL and OE GRG5 teratomas. Mean ± SD of n = 8 independent teratomas. *P < 0.05 (**e**) Pictures and quantification of immunohistological staining for the proliferation marker Ki67 in CONTROL and OE GRG5 teratoma cryosections. DAB was used as chromogen and Hematoxylin as counterstain (x100). Mean + SD of n = 2 independent experiments. (**f**) H&E stained cryosections of CONTROL and OE GRG5 teratomas showing hemorrhagic areas (white arrowhead) (Scale bar, 50 μm) Quantification of the volume of the hemmorhagic region in CONTROL and OE GRG5 teratomas. Mean + SD of n = 3 independent experiments. *P < 0.05 (**g**) H&E staining pictures showing CONTROL (black line) and OE GRG5 (black dots) teratoma borders. White arrow: Host muscle cells. (Scale bar, 100 μm) (**h**) Histological analysis with H&E and PAS-D staining of CONTROL and OE GRG5 teratoma cryosections presenting derivatives of the three primary germ layers. Black cross: Cartilage, Black star: Neuroepihelium, Black arrowhead: Columnal epithelium with goblet cells. (x100, x400) (**i**) Comparison of teratoma tissue composition based on analysis of H&E stained cryosections. Mean ± SD of n = 3 teratomas. *P < 0.05.

**Figure 3:**
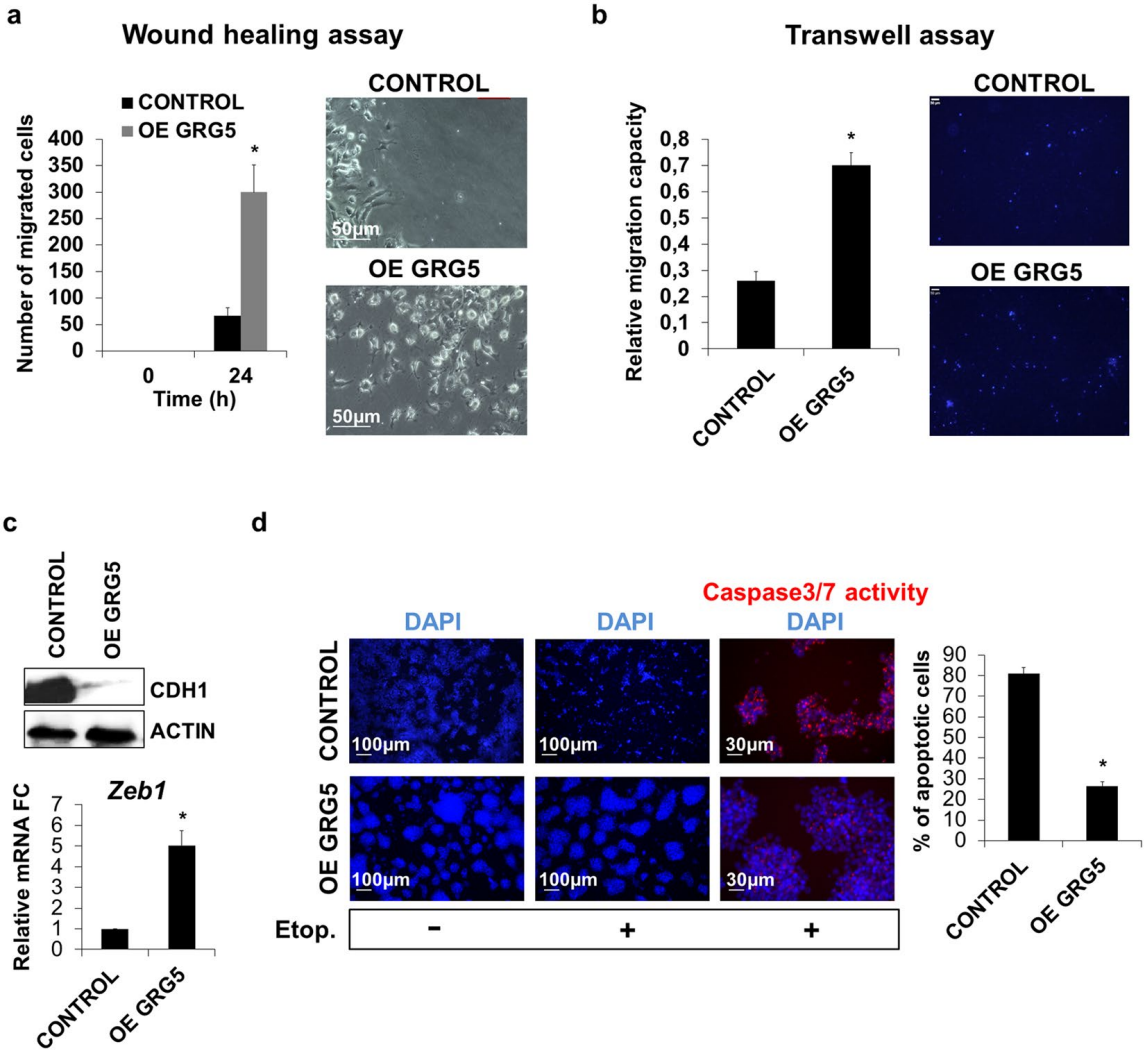
OE GRG5 ESCs present increased migration capacity and resistance to DNA damage. (**a**) Wound healing assay indicating increased migration capacity of OE GRG5 ESCs. Graph and phase contrast images presenting the number of migrated cells inside the gap area 24 h upon wound generation. Mean + SD of n = 3 independent experiments. *P < 0.05 (Scale bar, 50 μm) (**b**) Transwell assay showing the elevated migration capability of OE GRG5 ESCs. Quantification and photos of the DAPI stained migrated nuclei. Mean + SD of n = 3 independent experiments. *P < 0.05 (Scale bar, 100 μm) (**c**) Analysis of EMT related markers. Western blot for the epithelial marker CDH1 in CONTROL and OE GRG5 ESCs. qPCR analysis of the EMT mediator *Zeb1*. Mean + SD of n = 3 independent experiments. *P < 0.05 (**d**) Apoptosis assay demonstrating the resistance of OE GRG5 ESCs to DNA damage. Photos and quantification of the apoptotic CONTROL and OE GRG5 ESCs 22 h upon induction of DNA damage with 0.17 μM Etoposide treatment. *P < 0.05 (Scale bars, 100 μm and 30 μm).

Based on the results described above and a previous report that ectopic expression of GRG5 leads to embryonic lethality^41^, we sought to assess the impact of GRG5 overexpression on ESC phenotype. We generated and studied two stable GRG5-GFP ESC clones. Characteristic data obtained from clone #7 (OE GRG5 ESCs) are presented in Figs 2 and 3.

The protein level of GRG5 in the overexpressing clones was three times higher as compared to the control cells (Supplementary Fig. S3a). Forced expression of GRG5 resulted in increased protein levels of key pluripotency factors (Fig. 2a) and higher proliferative potential as revealed by analysis of RB phosphorylation level (Fig. 2b) and growth rate evaluation (Fig. 2c). To test whether this phenotype persists upon induction of ESC specification, we performed a series of *in vitro* differentiation through EB formation. OE GRG5 ESCs generated EBs with larger size compared to CONTROL (Supplementary Fig. S3b,c). In accordance, western blot analysis showed increased CCND1 expression and persistence of inactive phosphorylated form of RB protein (ppRB) during differentiation (Supplementary Fig. S3d). Additionally, we observed belated decrease in OCT4 protein levels indicating delayed differentiation (Supplementary Fig. S3d). These findings confirm the active role of GRG5 in self-renewal regulation and highlight a previously unknown growth-promoting activity.

To study the functional significance of GRG5 overexpression *in vivo*, we injected ESCs intramuscularly in immunocompromized mice. OE GRG5 ESCs formed teratomas of larger size in comparison to CONTROL (Fig. 2d). Immunohistological staining for the proliferation marker Ki67 showed increased proliferation in regions rich in blastema (Fig. 2e). Moreover, OE GRG5 teratomas showed malignant behavior manifested by heterogeneous morphology, wide hemorrhagic areas (Fig. 2f) and strong invasion capacity penetrating the adjacent muscle tissue (Fig. 2g). On the contrary, teratomas derived from the CONTROL ESCs were characterized by well-defined borders. Evaluation of teratoma tissue composition with histological analysis and immunohistochemistry for representative germ layer markers revealed greatly reduced presence of endodermal origin tissues and increased percentage of blastema and neuroectoderm in case of GRG5 overexpression (Fig. 2h, i, Supplementary Fig. S3e).

Finally, we studied the effect of direct intraperitoneal injection of CONTROL and OE GRG5 ESCs. Interestingly, none of the mice injected with CONTROL ESCs developed teratomas up to 35 days post-inoculation, whereas mice injected with OE GRG5 ESCs developed several tumors with widespread distribution (Supplementary Fig. S3f). Noteworthy, tetatomas developed not only as attached to the organs but also as nodes within the liver and lung tissue suggesting a metastatic process.

To exclude the possibility of chromosomal abnormalities that are often linked with increased ESC tumorigenicity^42^ we performed karyotype analysis which did not show differences in OE GRG5 ESCs (Supplementary Fig. S3g). Notably, similar results were obtained using a different OE GRG5 ESC clone #1 (Supplementary Fig. S4).

These findings prompted us to study GRG5 overexpressing ESCs in more detail for additional tumor cell like properties. We used *in vitro* wound healing assays to analyze their migration potential upon induction of differentiation with RA treatment. These experiments showed that GRG5 enhanced cell motility as measured by a 24 h scratch distance (Fig. 3a). The increased migratory response was also confirmed in a transwell migration assay. The percentage of cells that migrated toward a chemo-attractant gradient was measured 16 h upon plating and was higher in case of OE GRG5 ESCs (Fig. 3b).

As GO term analysis showed enrichment of cell motility and migration-related processes in KD GRG5 cells, that are often linked with epithelial-mesenchymal transition (EMT), we next checked expression of epithelial markers. Indeed, in OE GRG5 ESCs the epithelial marker CDH1 was significantly decreased (Fig. 3c) while the expression of the EMT mediator Zeb1 was increased (Fig. 3c).

Finally, we set out to test whether GRG5 levels affect cellular response to death signals. ESCs prevent the accumulation of mutations and preserve their genetic integrity by displaying hypersensitivity to DNA damage^43^. Induction of DNA damage using Etoposide treatment and consequent detection of CASPASE3/7 activity showed that OE GRG5 ESCs exhibit apoptosis resistance (Fig. 3d).

In summary, our *in vivo* and *in vitro* experiments show that overexpression of GRG5 in ESCs increases cell growth along with their tumorigenic potential and leads to the generation of malignant teratomas highlighting its pro-oncogenic potential.

### GRG5 promotes ESC neural specification by suppressing Wnt and Bmp signaling

A number of studies have implicated Groucho proteins in neurogenesis regulation^44–47^. Our EB analysis already associated the absence of GRG5 with increased expression of mesendodermal genes and decreased levels of neuroectodermal markers (Fig. 1f). These observations along with the enrichment of the anti-neurogenic Wnt and Bmp pathways^48^ upon GRG5 depletion (Fig. 1e, Table 1) suggest that GRG5 may also be involved in ESC neuroectodermal specification.

**Figure 4:**
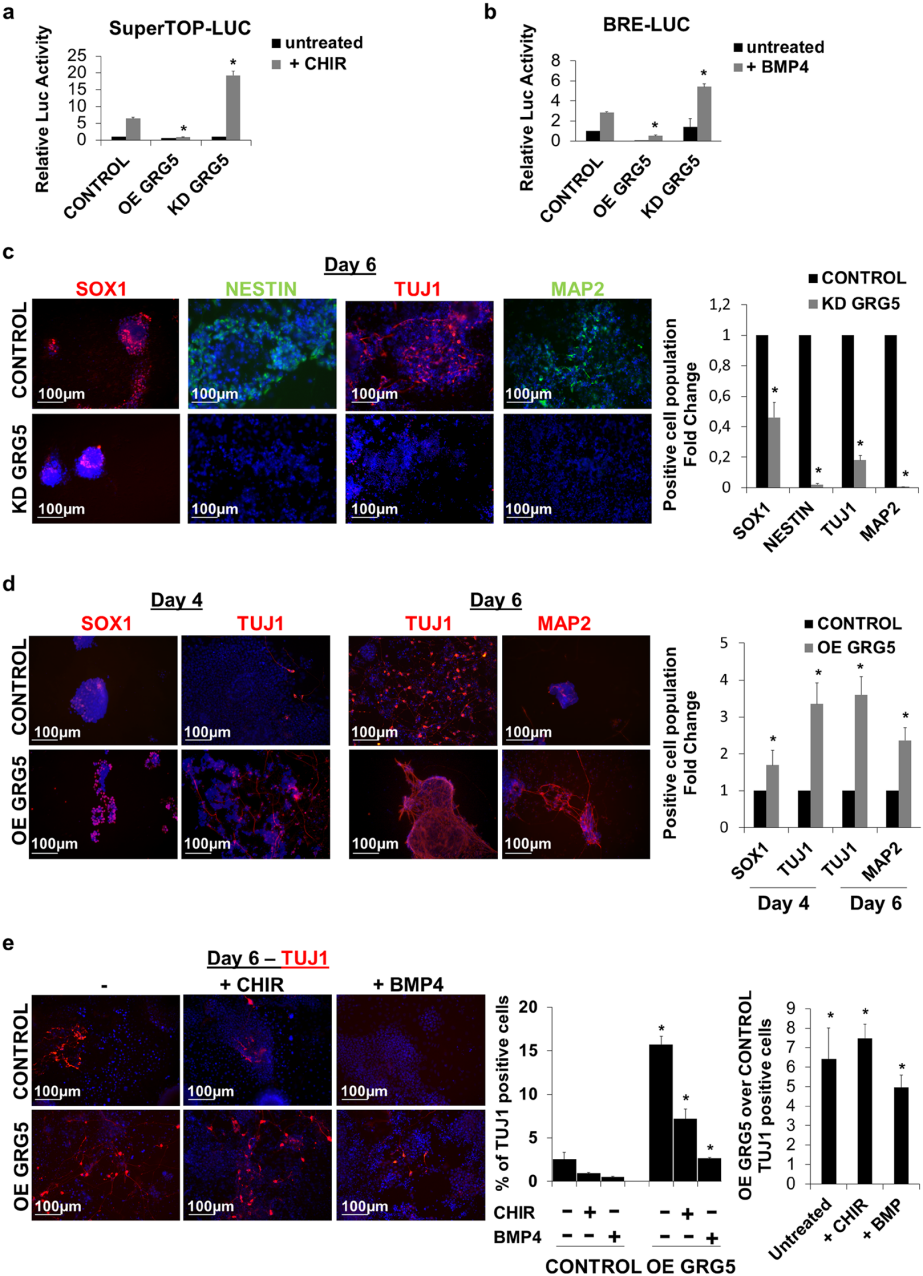
GRG5 regulates ESC competence for neuroectodermal commitment by suppressing Wnt and Bmp signaling. (**a**,**b**) Luciferase assays using the SuperTOP-LUC (**a**) and BRE-LUC (**b**) reporter plasmids to monitor Wnt and Bmp signaling respectively in CONTROL, OE GRG5 and KD GRG5 ESCs. Mean + SD of n = 3 independent experiments. *P < 0.05 (**c**) CONTROL and KD GRG5 ESCs were cultured in neural differentiation conditions for 6 days. Immunofluorescence photos and quantification of cells expressing neural progenitor (SOX1, NESTIN) and neuronal (TUJ1, MAP2) markers at Day6. (Scale bar, 100 μm) Mean + SD of n = 4 independent experiments. *P < 0.05 (**d**) CONTROL and OE GRG5 ESCs were differentiated towards neural lineage for 6 days. Photos and quantification of cell immunofluorescence for neural progenitor (SOX1) and neuronal (TUJ1, MAP2) markers at days 4 and 6. (Scale bar, 100 μm) Mean + SD of n = 4 independent experiments. *P < 0.05 (**e**) Wnt and Bmp signaling pathways were stimulated using CHIR and BMP4 treatment in CONTROL and OE GRG5 ESCs, 24 h before induction of neural differentiation. Immunofluorescence analysis and quantification of TUJ1 positive neurons at Day6 of the neural specification procedure. (Scale bar, 100 μm) Mean + SD of n = 3 independent experiments. *P < 0.05.

We performed luciferase assays and confirmed that GRG5 overexpression suppresses both Wnt and Bmp signaling. CONTROL, KD and OE GRG5 ESCs were transfected with the reporter plasmid SuperTOP-LUC for the Wnt pathway and BRE-LUC for the Bmp pathway. Cells were stimulated with CHIR and BMP4, respectively (Fig. 4a,b). Accordingly, expression analysis of the Wnt downstream targets *T-brac* and *Axin2*, as well as Bmp targets *Id1* and *Id3* confirmed the reduction in the activities of the two pathways upon GRG5 gain of function (Supplementary Fig. S5a,b). To date, no correlation between GRG5 and Bmp signaling has been shown. To elucidate the mechanism behind this effect,we performed co-immunoprecipitation assays in HEK293 cells and found a physical interaction between GRG5 and the Bmp pathway mediators SMAD1 and SMAD5, but not SMAD8 (Supplementary Fig. S5c). We next employed the GAL4-UAS luciferase system to identify the functional significance of the interaction between GRG5 and SMAD1. The activity of the luciferase reporter was tested upon expression of a fused SMAD1-GAL4 protein in the presence/absence of GRG5. Overexpression of GRG5 inhibited the transcriptional activity of SMAD1 (Supplementary Fig. S5d). These data suggest a repressive mechanism of action in which GRG5 interacts with SMADs and modulates their transcriptional activity. In view of these findings and the positive regulation of the neuroectoderm lineage-specifier SOX2 by GRG5 (Figs 1b and 2a)^48^, we hypothesized that the latter acts as a positive regulator of ESC neural specification.

We investigated ESC neuroectodermal specification using an established monolayer differentiation approach^49^, in which ESCs are cultured in serum free medium for 6 days. This approach leads to efficient generation of neural cell population and enables a better understanding of the differentiation process compared to the EB method. Characterization of GRG5 expression profile throughout the procedure revealed a transient drop upon ESC exit from the pluripotent state followed by an increase during neuronal differentiation (Supplementary Fig. S5e). We then examined the neural differentiation capacity of KD GRG5 ESCs using immunostaining against the neural progenitor cell (NPC) markers SOX1 and NESTIN, as well as markers for the immature (TUJ1) and the mature (MAP2) neuronal cells. GRG5 loss of function severely reduced the percentage of both committed NPCs and postmitotic neurons indicating that GRG5 is required for the successful ESC neuroectodermal commitment (Fig. 4c). In contrast, overexpression of GRG5 accelerated neural commitment, yielding higher percentage of both SOX1+ NPCs and TUJ1+ neurons by Day 4 (Fig. 4d). The percentage of neurons remained significantly higher at Day 6 in comparison to CONTROL ESCs (Fig. 4d).

To examine whether GRG5 repressive activity over Wnt and Bmp signaling is associated with its role in neuronal differentiation, we stimulated each pathway 24 h before induction of differentiation. Quantification of TUJ1+ neurons at Day 6 showed that the activation of both Wnt and Bmp in CONTROL ESCs severely reduced the percentage of neural cells. In contrast, Wnt and Bmp activation had minor effects in OE GRG5 ESCs (Fig. 4e). Overall, our analysis shows that GRG5 serves as a critical determinant that promotes ESC neuroectodermal specification through inhibition of Wnt and Bmp signaling.

### GRG5 is required for direct neuronal conversion of fibroblasts

**Figure 5:**
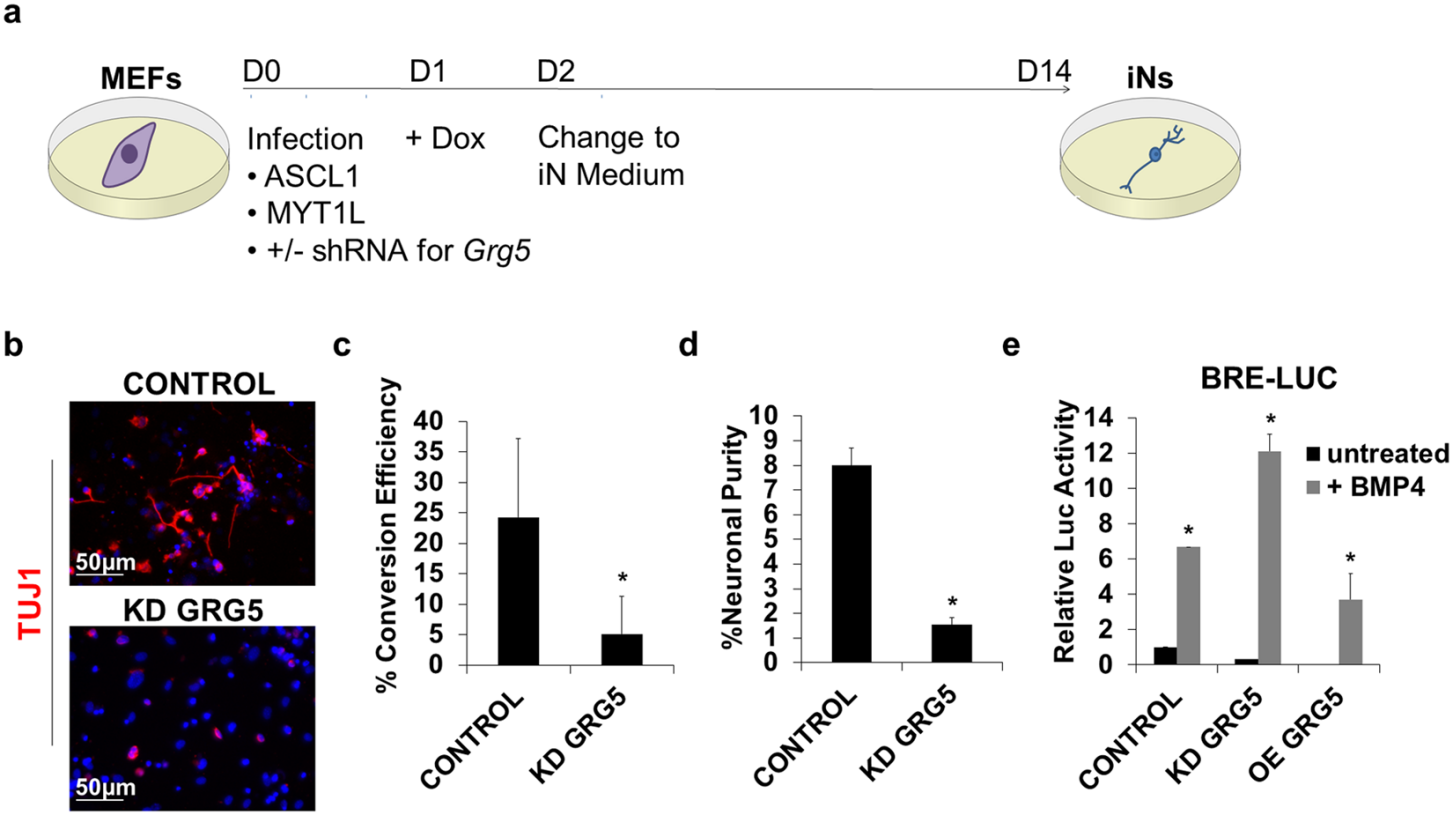
GRG5 is essential for the direct conversion of MEFs to induced neurons. (**a**) Schematic representation of the experimental process for the direct neuronal conversion of MEFs. MEFs were infected with inducible lentiviruses expressing the proneural factors ASCL1 and MYT1L. Silencing of GRG5 expression was achieved using specific shRNA. From Day 2 post-infection cells were cultured in neuronal promoting conditions (iN medium). On Day14 the generation of induced neuronal cells (iNs) was evaluated. (**b**) Representative immunofluorescence photos showing TUJ1 positive neuronal cells derived from CONTROL and KD GRG5 MEFs at Day 14. (Scale bar, 50 μm) (**c**) Conversion efficiency estimated by dividing the number of TUJ1 positive cells at Day14 with the number of the plated MEFs at Day 0. Mean + SD of n = 3 independent experiments. *P < 0.05 (**d**) Neuronal purity defined as the number of TUJ1 positive cells at Day14 divided by the total number of DAPI positive cells at Day 14. Mean + SD of n = 3 independent experiments. *P < 0.05 (**e**) Luciferase assay monitoring the activity of BMP signaling using the BRE-LUC reporter plasmid in MEFs upon transient silencing or over-expression of GRG5. Mean + SD of n = 3 independent experiments. *P < 0.05.

To further study the role of GRG5 in neural lineage commitment we turned to a direct neuronal reprogramming system and tested whether GRG5 was required for induced Neurons (iN) generation. To this end we used lentiviruses to ectopically express the key reprogramming factors ASCL1 and MYT1L in combination with control (CONTROL) or Grg5 specific (KD GRG5) shRNAs in mouse embryonic fibroblasts (MEFs) (Fig. 5a). Conversion efficiency was evaluated 14 days past infection with immunostaining by counting the numbers of TUJ1+ neurons. Loss of GRG5 almost completely abrogated reprogramming of MEFs to iNs (Fig. 5b). Both conversion efficiency (Fig. 5c) and neuronal purity (Fig. 5d) were severely decreased in the absence of GRG5 indicating that GRG5 activity is necessary for successful conversion of fibroblasts to induced neurons.

Taking into consideration the inhibitory effect of SMAD signaling on iN generation^14,50^ and our finding that GRG5 impairs Bmp pathway activity in ESCs, we tested whether GRG5 has analogous repressive role in MEFs. Luciferase assay after transient silencing or overexpression of GRG5 showed that Bmp pathway activity was significantly augmented or decreased upon KD or OE of GRG5, respectively (Fig. 5e). These results show that lack of GRG5 increases Bmp signaling, thereby contributing to the impaired ability of KD GRG5 MEFs to generate iNs.

### Maintenance of embryonic NSC identity entails GRG5 function

**Figure 6:**
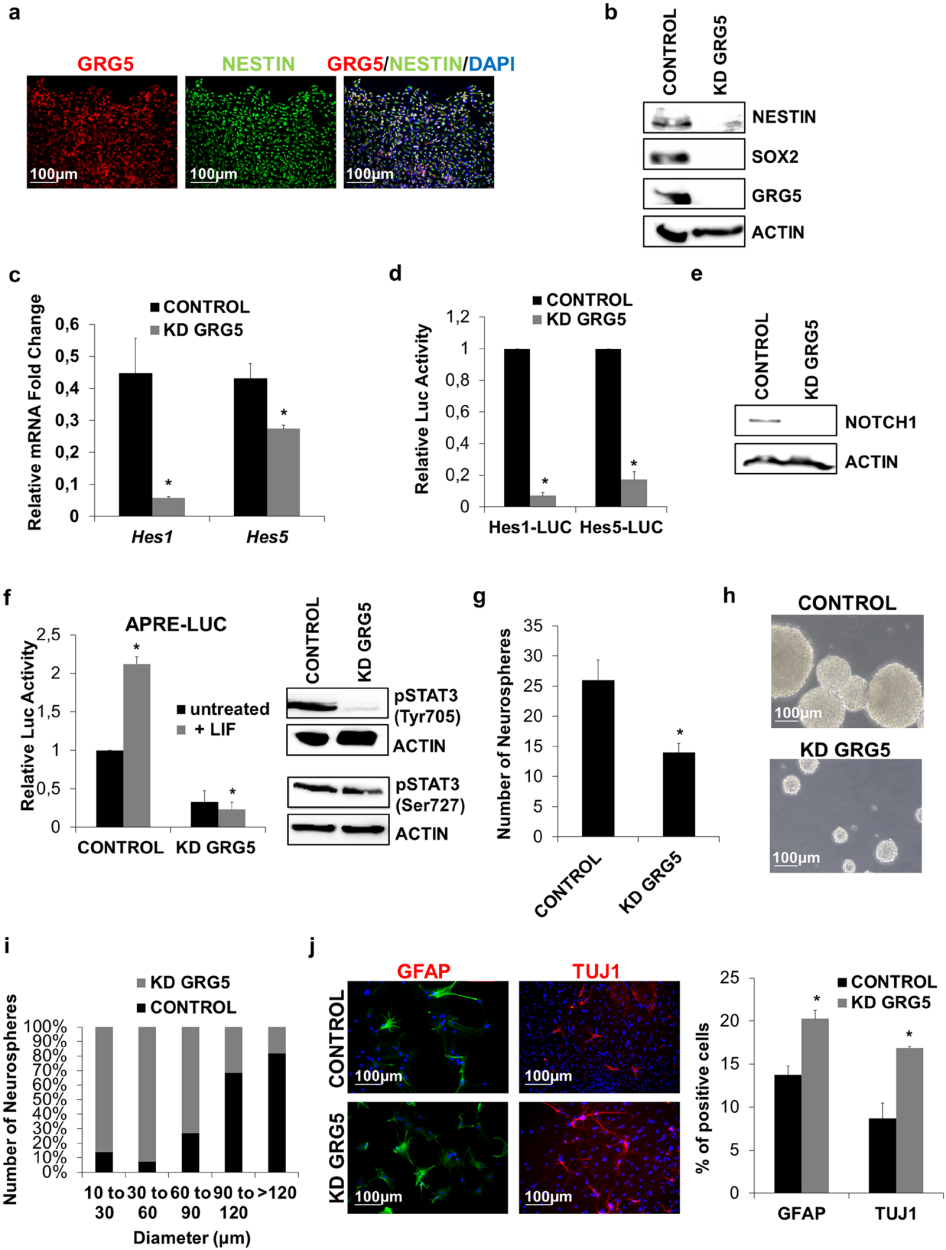
GRG5 severely contributes to embryonic NSC identity maintenance. (**a**) Immunofluorescence staining for GRG5 and NESTIN in embryonic NSCs cultured in monolayer conditions. (Scale bar, 100 μm) (**b**) Protein levels of stemness factors in CONTROL and KD GRG5 NSCs. (**c**) Relative mRNA levels of the Notch mediators *Hes1* and *Hes5* in CONTROL and KD GRG5 NSCs. Mean + SD of n = 3 independent experiments. *P < 0.05 (**d**) Notch signaling is down-regulated in the absence of GRG5. Luciferase assay monitoring the activity of Notch pathway in CONTROL and KD GRG5 NSCs. Data are shown as Mean + SD of n = 3 independent experiments. *P < 0.05. (**e**) Protein levels of NOTCH1 receptor in CONTROL and KD GRG5 NPCs. (**f**) Luciferase assay showing decreased STAT3 binding activity and western blot showing reduced protein level of p-STAT3 (Tyr705 and Ser727) in KD GRG5 NSCs. Mean + SD of n = 3 independent experiments. *P < 0.05 (**g**) Neurosphere forming assay comparing CONTROL and KD GRG5 NSCs. Six days upon cell culture in proliferation medium, the total number of the formed neurospheres was counted. Mean + SD of n = 3 independent experiments. *P < 0.05 h) Phase-contrast images showing CONTROL and KD GRG5 neurospheres (Scale bar, 100 μm). (**i**) Comparison of CONTROL and KD GRG5 NSC proliferation capacity by measuring the size of the generated neurospheres. Mean of n = 3 independent experiments. (**j**) Loss of GRG5 facilitates NSC specification. Immunofluorescence staining for the astrocytic marker GFAP and the neuronal marker TUJ1 in CONTROL and KD GRG5 NSCs five days upon culture in differentiation medium (scale bar, 100 μm). Mean + SD of n = 3 independent experiments. *P < 0.05.

So far we have shown that GRG5 is required for the maintenance of ESC stemness as well as for neural fate specification. Based on these findings we reasoned that GRG5 could also be involved in NSC self-renewal. NSCs from embryonic mouse cortex E13.5 were isolated and were grown under neurosphere-forming conditions. Immunostaining showed that GRG5 was expressed in NESTIN+ NSCs (Fig. 6a), while Western blot analysis revealed comparable expression levels between NSCs and ESCs (Supplementary Fig. S6a).

To study the function of GRG5 in embryonic NSCs, we produced pools of cells infected with lentiviruses expressing control (CONTROL) or Grg5 specific (KD GRG5) shRNAs. GRG5 depletion resulted in a marked decrease in the expression of the NSC markers SOX2 and NESTIN (Fig. 6b).

Well documented is the role of Notch signaling through the activity of the bHLH HES factors in NSC self-renewal maintenance^51^. HES proteins are expressed upon Notch signaling activation and synergize with Groucho/TLE co-repressors for the suppression of neurogenic genes^46,51,52^. We thus examined the expression levels of *Hes1* and *Hes5* mRNAs (Fig. 6c) and the activity of their promoters (Fig. 6d) and found that they were reduced in the absence of GRG5. To determine the role of GRG5 in Notch pathway regulation we first evaluated the expression levels of NOTCH1 and found a strong reduction when GRG5 was knocked down (Fig. 6e). In addition, we tested whether GRG5 acts also downstream of NOTCH1. For this aim we co-transfected CONTROL and KD GRG5 ESCs with an NICD expressing plasmid and Hes-Luc reporters. The basal activities of both *Hes1* and *−5* promoters were reduced in the absence of GRG5 (GRG5 KD), in agreement with the previous results, however the addition of NICD restores them (Fig. S6b). Notably the fold induction exerted by NICD was similar in both cell types indicating that GRG5 may not be critical downstream of NOTCH1.

We also tested the activity of STAT3 signaling since both the canonical Jak/Stat3^53,54^ and the non-canonical Stat3/Hes3^55^ pathways have been linked to NSC maintenance. Luciferase assay and reduction of the pTyr705-STAT3 levels showed that the LIF/Jak/Stat3 pathway is defective in KD GRG5 NSCs (Fig. 6f). Noteworthy, there was also a reduction in the phosphorylation level of Ser727-STAT3 indicating that the non-canonical Stat3/Hes3 pathway is also affected (Fig. 6f).

Finally, in comparison to the CONTROL the number and the size of neurospheres formed by KD GRG5 NSCs was smaller suggesting that lack of GRG5 results in remarkable reduction of NSC self-renewal and proliferation capacity (Fig. 6g–i). Moreover, GRG5 loss of function makes NSCs more susceptible to differentiation as it resulted in higher percentage of both TUJ1+ neurons and GFAP+ astrocytes upon five days cell culture in differentiation promoting conditions (Fig. 6j).

Conversely, GRG5 overexpression in NSCs led to higher expression of the NSC key regulators SOX2 and NESTIN (Supplementary Fig. S6c). In addition, evaluation of neurosphere number and size revealed enhanced self-renewal capacity (Supplementary Fig. S6d) but no significant difference in cell growth rate (data not shown). Moreover, GRG5 gain of function stabilized NSC state as reduced numbers of TUJ1+ neurons and GFAP+ astrocytes were observed upon induction of cell differentiation (Supplementary Fig. S6e).

Taken together, these results show that GRG5 sustains the activity of Notch and Stat3 signaling and plays an essential role in NSC maintenance.

## Discussion

In this study, we have systematically analyzed the role of GRG5 in mouse ESCs and NSCs. Our results show that GRG5 is a regulator with pleiotropic role in stem cell biology: it acts as regulator of stem cell pluripotency that upon overexpression promotes oncogenicity but it also has a decisive role in neural fate specification.

In mouse ESCs, depletion of GRG5 deregulates the pluripotent state and de-represses a variety of developmental markers hinting ESC exit from pluripotency. TLE 3 and 4, two full length members of the GRG family were recently shown to be up-regulated when ESCs differentiate and to suppress the pluripotency network^56^. These data, taken together with the current study that shows an opposite expression mode for GRG5, suggest a potential antagonism between GRG5 and TLE 3/4. In agreement with this hypothesis both TLE 3 and TLE 4 are up-regulated in KD GRG5 cells (Table 1).

Our results point to a role of GRG5 in ESC maintenance, as its absence does not affect ESC viability or morphology. This is in accordance with the phenotype of Grg5^−/−^ (KO) mice^34^ which are viable possibly because of functional redundancy. Interestingly, GRG5 gain of function in ESCs leads to increased proliferative potential along with enhanced cell motility and resistance to DNA damage. Thus, regulated expression levels of GRG5 are required to maintain ESC stability: when it is ablated cells start to differentiate while its overexpression leads to cell transformation. Increased self-renewal of GRG5 overexpressing ESCs under differentiation conditions results in teratomas with malignant features when injected either intramuscularly or intraperitoneally, suggesting that GRG5 has a pro-oncogenic potential.

Malignant teratomas caused by deregulated expression of diverse genes were previously reported. Tet1-depleted ESCs produced teratomas with enhanced growth and wide hemorrhagic regions^57^, while teratomas from Lefty2 KD ESCs were characterized by high levels of OCT4 and SOX2 and contained massive expansions of immature neuroepithelia^58^. Smad3 knock out was also reported to cause ESC transformation and lead to malignant teratomas via the upregulation of RIF1^59^. Although the precise mechanism whereby GRG5 overexpression transforms ESCs is not known, this effect can be attributed to its cell growth and motility promoting activity as well as the up-regulation of factors that are already linked with tumorigenesis including SOX2 and NANOG^60^. Noteworthy, the oncogenic activity of GRG5 has been also reported in AML where it promotes the self-renewal of hematopoietic stem cells^30^. It is possible that the central regulatory role of GRG5 on ESCs state underlines the embryonic lethality of overexpressing transgenic mice^41^.

Beside the involvement of GRG5 in the regulation of pluripotency, our data show that it is also involved in ESC lineage specification. Our genome-wide expression analysis of ESCs showed that both Wnt and BMP pathway activities are increased in the absence of GRG5 pointing to a preference of meso- and endodermal lineages with a concomitant repression of the ectoderm. Noteworthy, Tcf7l1 (formerly known as Tcf3), a negative regulator of pluripotency and a primitive streak activator^61^ is highly increased in the absence of GRG5. In agreement, differentiation assays through EBs formation indicated that GRG5 is required for the neuroectodermal commitment of ESCs. Moreover, using a monolayer differentiation approach we showed that GRG5 promotes neuronal commitment through suppression of the anti-neurogenic Wnt and Bmp pathways.

GRG5 was previously found to antagonize Wnt/ β-catenin signaling in human cells and zebrafish embryos serving as a TCF4/Tcf7l2 corepressor^62^. In this study, we link for the first time GRG5 with the Bmp cascade and provide a mechanism whereby GRG5 suppresses Bmp signaling by inhibiting the trans-activation potential of SMADs via direct interaction. Furthermore, we show that the depletion of GRG5 severely impedes direct neuronal conversion of fibroblasts and attribute this function, at least partially, to the up-regulation of the BMP pathway that is known to inhibit the transdifferentiation process^14,50^.

Considering the conserved role of Groucho proteins in neurogenesis^45^, we also explored the function of GRG5 in embryonic NSC maintenance and found that it promotes their self-renewal. GRG5 function is underlined by the up-regulation of SOX2 expression together with the potentiation of Notch/Hes and Stat3 signaling pathways^53–56^. A functional interaction between GRG5 and SOX2 similar to the one described recently in human NSCs^63^ cannot be excluded. In this context, the role of GRG5 resembles that of the anti-neurogenic function of GRG1 in neural progenitor cells^46^, even though GRG5 has been reported to antagonize GRG1 in other systems. Although there is no report for neurological disorders in GRG5 KO mice, our data suggest that further examination of their brain development may reveal important information.

In summary, we demonstrate that GRG5 is a multifunctional regulator of ESCs and NSCs. Dissecting the mechanisms of cell transformation by GRG5 overexpression would have an impact in understanding oncogenesis during teratoma formation and the association of GRG5/AES with human malignancies. Furthermore, the observed functions of GRG5 in neuronal specification support further investigation of GRG5 implication in brain development and disease.

## Methods

All methods were performed in accordance with the IMBB guidelines and safety regulations.

### ESC culture and differentiation

Feeder-independent CGR8 murine ESCs were cultured on 0.2% gelatin in DMEM medium (GIBCO) supplemented with 15% Fetal Bovine Serum (FBS) (GIBCO), 0.2 mM β-mercaptoethanol (Applichem), 2 mM L-glutamine (GIBCO), 1× MEM nonessential amino acids (GIBCO), and 500 U/ml LIF (ESGRO/Millipore). EBs formation was performed using the hanging drop method as previously described by Hadjimichael and coworkers^64^. For ESC neuroectodermal specification cells were cultured at low density in serum free medium (N2B27) following the method outlined by Ying *et al*.^49^. Cell treatment with 2 mM CHIR (Selleckchem) and 80 ng/ml BMP4 (R&D) was performed 24 hr prior induction of differentiation in media deprived of LIF.

### Generation of KD and OE GRG5 ESC lines

Silencing of GRG5 expression was achieved using two independent Grg5 specific shRNA-Plko.1 lentiviral vectors (REF Sigma Aldrich, SHCLNV-NM_010347, TRCN0000097716, TRCN0000097717). As control an empty shRNA-pLKO.1 lentiviral vector was used. CGR8 cells were grown to 80% confluency and infected with lenti-viruses carrying the empty or *Grg5* specific shRNA-pLKO.1 vector, for 72 h. Pools of infected cells were selected with puromycin (2 μg/ml) and GRG5 silencing was examined at protein and mRNA level. Both knockdown cell lines presented similar efficiency of GRG5 depletion and comparable behavior. In the main figures we present data obtained with TRCN0000097716 Grg5 shRNA.

GRG5 OE ESC lines were generated upon ESC transfection with a GRG5-GFP expression vector and selection with G418 (400 μg/ml). Sequencing of the GRG5-GFP vector is provided in Table S1. An empty GFP expression vector was used as control. Out of many resistant clones two distinct clones (#1 and #7) were further studied and presented similar behavior both *in vitro* and *in vivo*.

### RNA-seq analysis

RNA samples were isolated in duplicates from WT and KD GRG5 ESCs and sequenced in Ion Torrent platform. Quality control of the sequence output was conducted with FASTQC. After inspection of each file, all the reads were trimmed using Trimmomatic^65^ in order to increase the overall quality across read positions. Reads of 30 nt length or less, were cut out. Afterwards the first 30 bases of each read were trimmed. Finally reads that were longer than 200 nt were truncated by trimming their 3′ends. Reads were then mapped against the mm10 reference genome with the aid of the STAR (Spliced Transcripts Alignnment to a Reference) aligner^66^ through the Galaxy interface (usegalaxy.org)^67^. Gene quantification was performed with FeatureCounts^68^ and differential gene expression was calculated with DeSeq.2^69^. All the algorithms that were used on Galaxy were executed with their default parameters. The final output of this procedure is a list of genes, with each gene followed by a log2FC value and a p-value. Genes that were not expressed in either condition were filtered out of the final list. Differentially expressed genes (DEG) were qualified as such on the basis of absolute log2(FoldChange) ≥ 0.65 coupled with a p-value ≤ 0.05.

### Overlap analysis for gene lists

In order to determine whether the knockdown of OCT4 on embryonic stem cells has an effect on the same subset of genes that are differentially expressed in our KD GRG5 ESCs experiment, OCT4 Knockdown datasets were retrieved from GEO (Platform ID: GPL1261)^70^. Using GEO2R, a list of relative expression values was extracted (OCT4 KD ESCs versus E14TG2A, wild type ESCs). Afterwards the same cut-offs used in our GRG5 KD analysis were applied (absolute log2 (FoldChange) ≥ 0.65 and p-value ≤ 0.05) generating a list of differentially expressed genes for the OCT4 KD dataset. The two lists were split into over-expressed and under-expressed genes. In order to assess the degree of overlap with our GRG5 DEG, two Jaccard indexes were calculated, one for each of the over- and under-expressed gene lists. The Jaccard index is calculated as the number of common genes between two lists divided by the total number of different genes found between them, that is their intersection over their union. In order to assess the significance of the calculated Jaccard indexes a permutation analysis was carried out. One thousand randomized gene lists were thus generated, with the same size as the GRG5 KD DEG using a custom Perl script and the 1000 corresponding Jaccard indexes were calculated for a) over-expressed and b) under-expressed genes. Having calculated the mean and standard deviation of the Jaccard indices of the permutated lists, it was assessed whether the initial, observed Jaccard index, was similar to that distribution on the basis of a z-score (which equals to the observed value minus the mean, divided by the standard deviation).

### Co-Immunoprecipitation Assay

To detect GRG5 protein interactors in ESCs immunoprecipitation of interacting protein complexes was performed using stably expressing GRG5-GFP and control GFP ESC lines. Cells were lysed in EBC buffer (50 mM Tris PH 8, 170 mM NaCl, 0.5% NP40, 50 mM NaF) containing 1 mM PMSF and protease inhibitors. 200 μg whole cell extracts were incubated with primary antibody overnight. The following day, whole cell extract of GRG5-GFP ESC was incubated with GFP-Trap MA beads (Chromotek) for 2 h at 4oC. GFP ESC lysate was used as negative control. For washing and elution steps manufacturer’s instructions was followed. SDS sample buffer was added and immunoprecipitated proteins were resolved by SDS-PAGE, followed by Western blotting as previously described^71^.

To examine whether GRG5 interacts with SMADs 1, 5 and 8, HEK293 cells were transfected with GRG5-GFP and pCDNA3-6myc-SMAD1, SMAD5 or SMAD8 expression vectors. HEK293 transfected with GRG5-GFP and an empty pCDNA3-6myc were used as negative control. Cells were lysed as described above and 200 μg whole cell extract was immunoprecipitated with anti-MYC and immunoblotted with anti-GRG5.

### Antibodies

Proteins were detected with primary antibodies presented in Table S1.

### Quantitative RT-PCR

Total RNA was extracted using TRIzol reagent (Thermo). 2 μg RNA was reversely transcribed to cDNA by M-MuLV Reverse Transcriptase (NEBiolabs) supplemented with RNase inhibitor (NEBiolabs) according to the manufacturer’s instructions. Quantitative RT-PCR was carried out using SYBR Green (Thermo) based detection and gene expression was normalized to β-Actin. The primers are presented in Table S2.

### Luciferase Assay

Cells were transfected with APRE‐Luc, SuperTOP-Luc or BRE‐Luc reporter plasmids using Lipofectamine 2000 (Thermo Fisher Scientific) and were stimulated 24 h later using 500 units/ml LIF (ESGRO/Millipore), 2 μM CHIR99021 (Selleckchem) and 80 ng/ml BMP4 (R&D) respectively. Hes1-Luc and Hes5-Luc reporters were used to measure Notch signaling activity. Cells were co-transfected with NICD expression plasmid to activate Notch signaling. Cells were co-transfected with the 5xGAL4UAS luciferase reporter and a SMAD1-GAL4 expression vector to detect the transcriptional activity of SMAD1. Luciferase activity was assayed 48 h upon transfection and was normalized using β-galactosidase reporter assay.

### Teratoma formation and evaluation

ESCs were trypsinized and injected intramuscularly or intraperitoneal (2 × 10^6^ cells in volume of 100 μl 1XPBS per mouse) in male NOD/SCID gamma mice. Teratomas were isolated 25 or 35 days upon intramuscular or intraperitoneal cell inoculation respectively and were further studied.

### ESC karyotyping

For karyotype analysis ESCs were seeded on gelatin in ESC medium. When cells became 70% confluent the medium was changed and 2 h later they were treated with 20 ng/ml Demecolcine (Sigma D6279) to synchronize cells in metaphase. After 1 h cells were trypsinized, incubated with hypotonic solution for 6 min, fixed (3Methanol: 1Acetic Acid) and dropped on acid washed slides. Cell nuclei were visualized with DAPI staining.

### Histological and Immunohistochemical analyses

Teratomas were snap frozen or fixed in 10% formalin solution and processed for routine histological examination. Formalin-fixed, paraffin-embedded tissue sections were stained with H & E, and PAS-D. Immunohistochemistry was performed as previously described^72^. In brief, after deparaffinization, rehydration and heat-induced epitope retrieval, slides were incubated with primary antibodies. Detection of the immunoreaction was performed using the EnVisionFlex kit (Dako) and 3,3-diaminobenzidine/H2O2 (Dako) and Haematoxylin was used as counterstain.

### Wound healing assay

The migration assay was performed using the method described by Li et coworkers^59^. Briefly, 3 × 105 ESCs were seeded in a 35 mm cell culture dish, in ESC culture medium. Two days later LIF was replaced by 1 μM Retinoic Acid (Sigma). 24 h upon RA treatment scratches were generated and detached cells were removed with PBS washes. 0 h and 24 h upon wound generation images were taken and the number of migrated cells was counted.

### Transwell migration assay

ESCs were detached by accutase (SIGMA) treatment. Following centrifugation cells were suspended in 1% FBS ESC culture media containing 0.1% BSA (GIBCO) and 1 μM Retinoic Acid (SIGMA) and plated at 0.15 × 10^6^ cells/ml on a 8 μm pore size membrane. The lower compartment was filled with 10% FBS containing ESC media. After 24 hr incubation the transwell insert was removed and placed into 4% paraformaldehyde for 10 min. Cells were washed with dΗ2Ο to remove paraformaldehyde. Using a cotton swap cells were scraped off the top of the transwell insert. Afterwards, cells were treated with a staining solution of 10 μg/ml DAPI, 0.5% Triton X-100 in 1x PBS, for 10–15 min. Transwell insert was removed and washed with 1x PBS. Migrated cells were visualized and counted under the microscope.

### Apoptosis Assay

ESCs were trypsinized and plated in ESC culture medium (3 × 10^5^ cells/9.5 cm^2^). The next day the medium was replaced by fresh ESC medium supplemented with 0.17 μm Etoposide. 22 h later cells apoptosis was evaluated using the Magic Red Caspase 3/7 Assay kit (ImmunoChemistry Technologies) according to the manufacturer’s instructions.

### Cell Immunofluorescence

Cells were fixed in 4% paraformaldehyde and permeabilized with 0.5% Triton X-100 in PBS for 15 min. Blocking was performed with 1% BSA in PBS for 1 h. Samples were then incubated with primary antibodies overnight. The antibodies used are described in the related section.

### Embryonic NSC isolation, culture and evaluation

Mouse NSCs were isolated from E13.5 embryonic brain. The cortex was, dissected, and incubated in basal medium (DMEM/F12 (GIBCO) supplemented with B27 (GIBCO), 2 mM GlutaMax (GIBCO), 15 mM Hepes (GIBCO) and 0.05 mg/ml Gentamicin (GIBCO)) at 37 °C for 20 min. Upon mechanical dissociation cells were cultured at 10 cells/μl in proliferation medium (basal medium supplemented with 20 ng/ml epidermal growth factor and 20 ng/ml basic fibroblast growth factor R&D Systems) as neurospheres. The medium was changed every three days and neurospheres were passaged using accutase (SIGMA) every six days.

For both KD and OE GRG5 pools of infected NSCs were used. To silence GRG5 expression, neurospheres were dissociated and infected with lentiviruses produced either by the empty shRNA-Plko.1 or Grg5 shRNA-Plko.1 vector (Sigma Aldrich, SHCLNV-NM_010347, TRCN0000097716) for 72 h. Selection of infected cells was based on puromycin (2 μg/ml) resistance. Accordingly, for GRG5 overexpression, cells were infected with lentiviruses GRG5-TetO-FUW and rtTA and selected with blasticidin 2 μg/ml. Sequences of the RT primers of the GRG5-TetO-FUW vector are provided in Table S2. To induce exogenous GRG5 expression 2 μg/ml doxycycline was used.

For self-renewal and proliferation capacity assessment, neurospheres were dissociated and 7 cells/μl were seeded in proliferation medium. Six days later both number and size of the formed neurospheres were measured. For the differentiation assay neurospheres were dissociated and plated on Matrigel coated surface in differentiation medium containing basal medium supplemented with N2 (GIBCO) and 1% FBS for five days. Differentiation efficiency was estimated with quantification of positive immunostained cells for neuronal and astrocyte specific markers.

### Neuronal conversion of MEFs

6 × 10^5^ MEFs were seeded on a Matrigel coated 24 well plate and 24 h later coinfected with the lentiviruses ASCL1-TetO-FEW, Myt1l-TetO-FEW, rtTA combined with pLKO.1 or pLKO.1 shRNA against Grg5 respectively (Sigma Aldrich, SHCLNV-NM_010347, TRCN0000097716). 24 h later, medium was replaced by fresh MEF medium (DMEM (GIBCO), 10% FBS (GIBCO), 2 mM L-glutamine (GIBCO)) supplemented with doxycycline (2 μg/ml). The next day, the medium was exchanged to iN medium (DMEM/F12 (GIBCO), supplemented with N2 (GIBCO), B27 (GIBCO), 2 mM GlutaMax (GIBCO), 0.05 mg/ml Gentamicin (GIBCO) and 2 μg/ml doxycycline)). The medium was changed every 3 days. Cells were immunostained for TUJ1 fourteen days later and efficiency was calculated. Conversion efficiency was estimated by dividing the average number of TUJ1+ cells with the number of the initially plated MEFs, whereas as neuronal purity is defined the average number of TUJ1+ cells per total number of DAPI+ cells at day 14.

### Statistical analysis

Student’s t-test was used for all statistical analyses. Statistical significance was defined as follows: *means p < 0.05. Values were presented as the mean ± SD.

### Ethical Approval for the Use of Animals

All experiments were conducted in accordance with the Laboratory Animal Care and Ethics Committee of IMBB. Animal work was approved by the IMBB Institutional Animal Care and Ethics Committee.

## Electronic supplementary material

Supplementary Information

Supplementary Dataset 1

Supplementary dataset 2

## Data Availability

The RNA-seq datashets generated and analyzed during the current study are available in the GEO repository with accession number GSE107068.
