# Supplementary material for: Groucho related gene 5 (GRG5) is involved in embryonic and neural stem cell state decisions
Source: Sci Rep. 2018 Sep 13;8:13790. doi: 10.1038/s41598-018-31696-9 (PMC6137157; doi:10.1038/s41598-018-31696-9)
Supplement: Supplementary file 1 — Supplementary Information [file 41598_2018_31696_MOESM1_ESM.pdf]

Supplementary Information

Groucho related gene 5 (GRG5) is involved in embryonic and neural stem cell state decisions

Konstantina Chanoumidou [1,2,3], Christiana Hadjimichael [2], Paraskevi Athanasouli [2,4], Henrik Ahlenius [3], Antonis Klonizakis [4], Christoforos Nikolaou [4], Elias Drakos [5], Antonis Kostouros [5], Irene Stratidaki [2], Maria Grigoriou [1], Androniki Kretsovali [2*]

[1.] Department of Molecular Biology and Genetics, Democritus University of Thrace, 68100 Alexandroupoli, Greece

[2.] Institute of Molecular Biology and Biotechnology, Foundation for Research and Technology-Hellas (FORTH), 70013 Heraklion, Crete, Greece

[3.] Lund Stem Cell Center, University Hospital, SE-221 84 Lund, Sweden

[4.] Department of Biology, University of Crete, 71409 Heraklion, Crete, Greece

[5.] School of Medicine, University of Crete, 71003 Heraklion, Crete, Greece

*Correspondence: kretsova@imbb.forth.gr

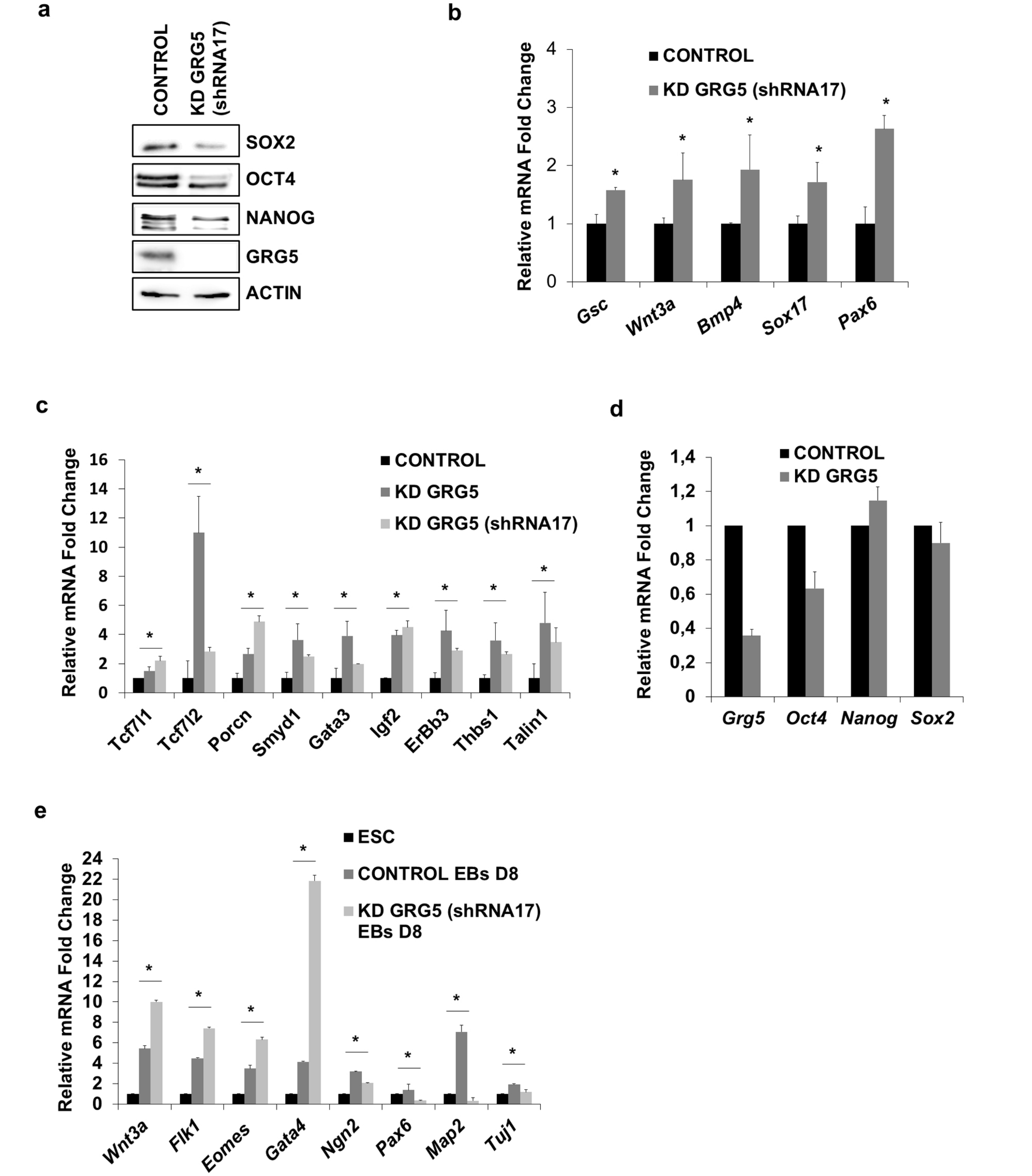

SI Figure 1: Analysis of KD GRG5 ESCs. In a, b, c and e are presented results obtained by the second KD GRG5 ESC line that was generated using an independent shRNA (shRNA17). (a) Protein levels of core pluripotency factors upon knockdown of GRG5. (b) Relative mRNA levels of differentiation markers in CONTROL and KD GRG5 ESCs. mean + SD of n=3 independent experiments. *P<0.05 **(c)** Validation of RNA-sequencing results. qPCR analysis of differentially expressed genes in CONTROL and both KD GRG5 ESC lines. mean + SD of n=3 independent experiments. *P<0.05. (d) Relative mRNA levels of pluripotency factors in CONTROL and KD GRG5 ESCs. mean + SD of n=3 independent experiments. (e) qPCR analysis of mesodermal (*Wnt3a, Flk1, Eomes*), endodermal (*Gata4*) and neuroectodermal (*Ngn2, Pax6, Map2, Tuj1*) markers upon differentiation of CONTROL and KD GRG5 ESCs through EBs formation at Day8. mean + SD of n=3 independent experiments. *P<0.05

a

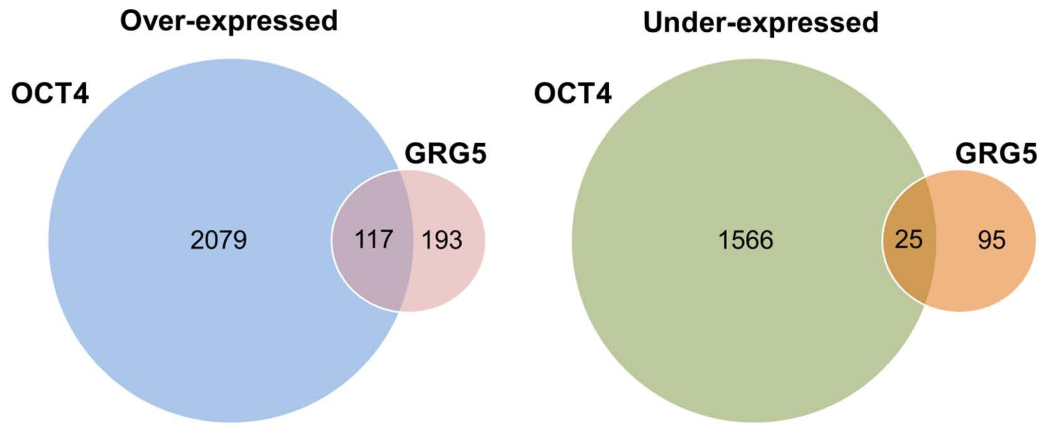

Comparison	Target genes Grg5/Oct4	Common Genes (intersect)	Unique Genes (union)	Jaccard Index (Intersect/)	Mean Jaccard Indexes (1000 permutations)	Z-scores	p-value
Over-expressed genes	310/2196	117	2389	0.049	0.011	18.47	1.80e-76
Under-expressed genes	120/1591	25	1686	0.0149	0.0043	6.87	3.21e-12

SI Figure 2: Comparison of GRG5 and OCT4 target genes. (a) Overlap of GRG5 and OCT4 target genes in ESCs. OCT4 knock down datasets were retrieved from GEO (Platform ID : GPL1261, Loh Yi et al). The degree of overlap for both over- and under-expressed genes was assessed by calculating the Jaccard similarity indexes.

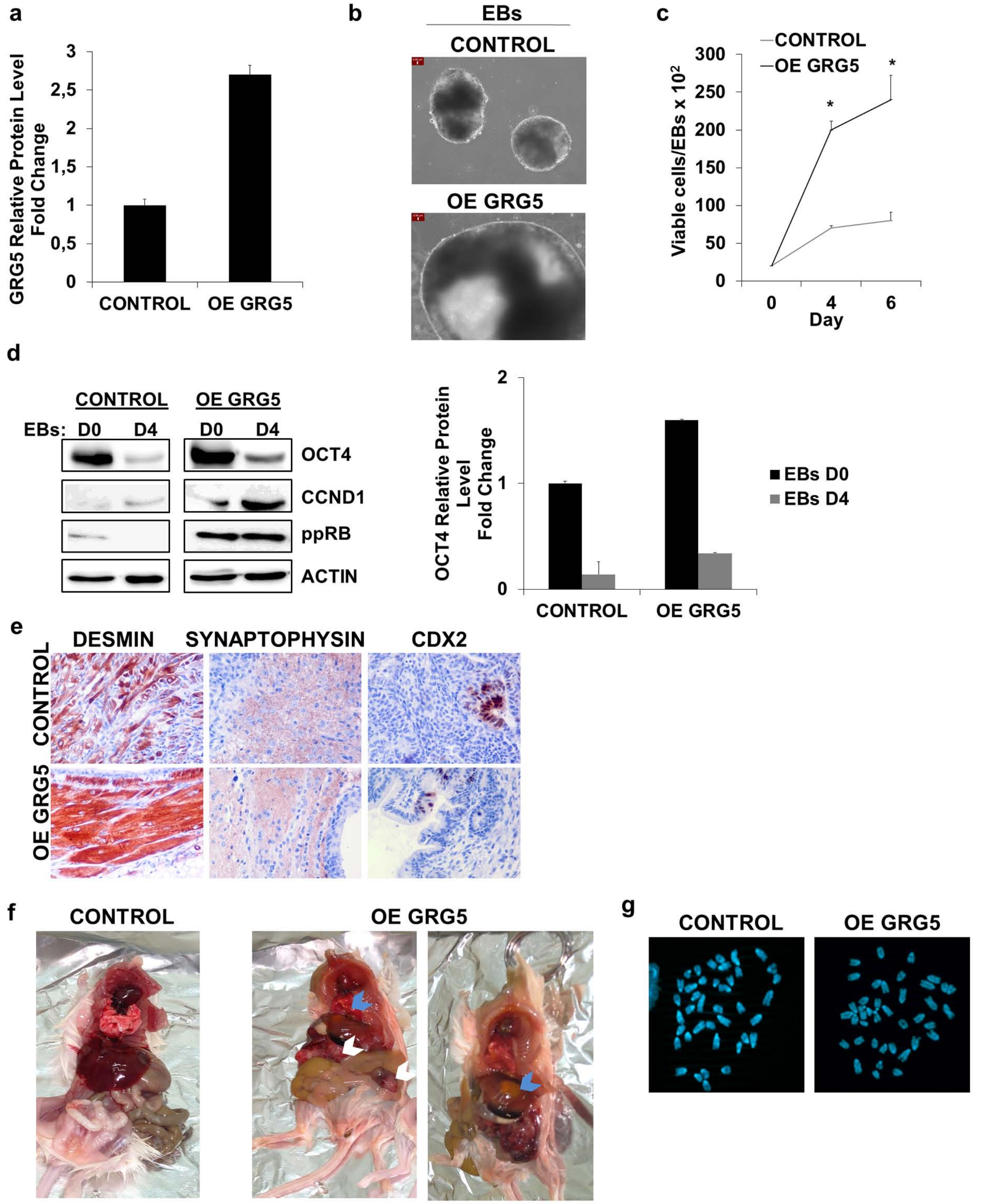

SI Figure 3: Impact of GRG5 over-expression on ESC proliferative and tumorigenic potential. (a) Relative protein level of GRG5 in CONTROL and OE GRG5 ESCs. (b) Phase/contrast images of CONTROL and OE GRG5 EBs at Day 6. (Scale bar, 0.06μm) (c) Growth curves of CONTROL and OE GRG5 EBs. EBs were dissociated and the total cell number per EB was counted. mean ± SD of n = 4 independent experiments. *P<0.05 (d) Protein levels of the pluripotency factor OCT4 and the proliferation markers CCND1 and ppRB in CONTROL and OE GRG5 EBs at Days 0 and 4. Quantification of OCT4 protein levels. (e) Immunohistological staining of CONTROL and OE GRG5 teratoma cryosections for representative markers of the three germ layers: DESMIN (mesoderm), SYNAPTOPHYSIN (ectoderm) and CDX2 (endoderm). DAB was used as chromogen and Hematoxylin as counterstain. (X100, X400) (f) OE GRG5 ESCs present enhanced tumorigenic capacity upon intraperitoneal inoculation in immunocompromised mice. White arrowhead: Teratoma. Blue arrowhead: Lung and liver with internal tumor nodes. (g) Karyotype analysis. Both CONTROL and OE GRG5 ESCs present intact chromosome number.

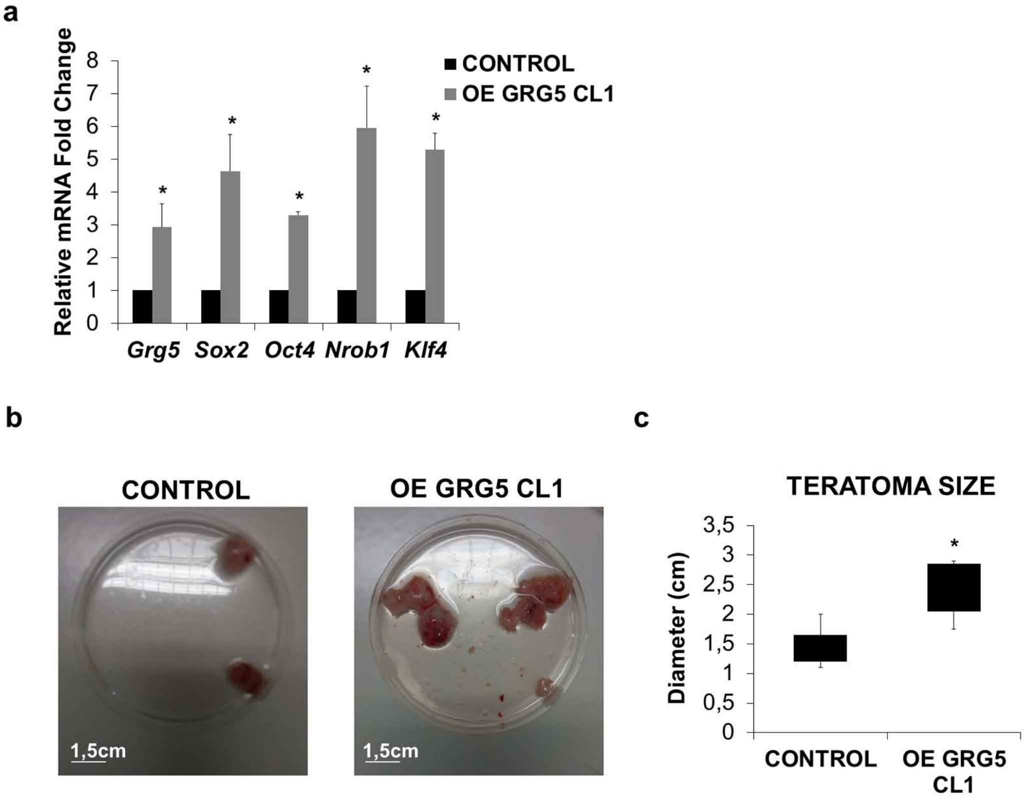

SI Figure 4: Analysis of a second OE GRG5 ESC clone (CL1). (a) Relative mRNA levels of pluripotency markers in CONTROL and OE GRG5 CL1 ESCs. mean + SD of n=3 independent experiments. . *P<0.05 (b) Photos showing teratomas derived from CONTROL and OE GRG5 CL1 ESCs (Scale bar, 1.5cm). (c) Box plot illustrating the average size of CONTROL and OE GRG5 CL1 teratomas. mean ± SD of n = 3 independent teratomas. *P<0.05

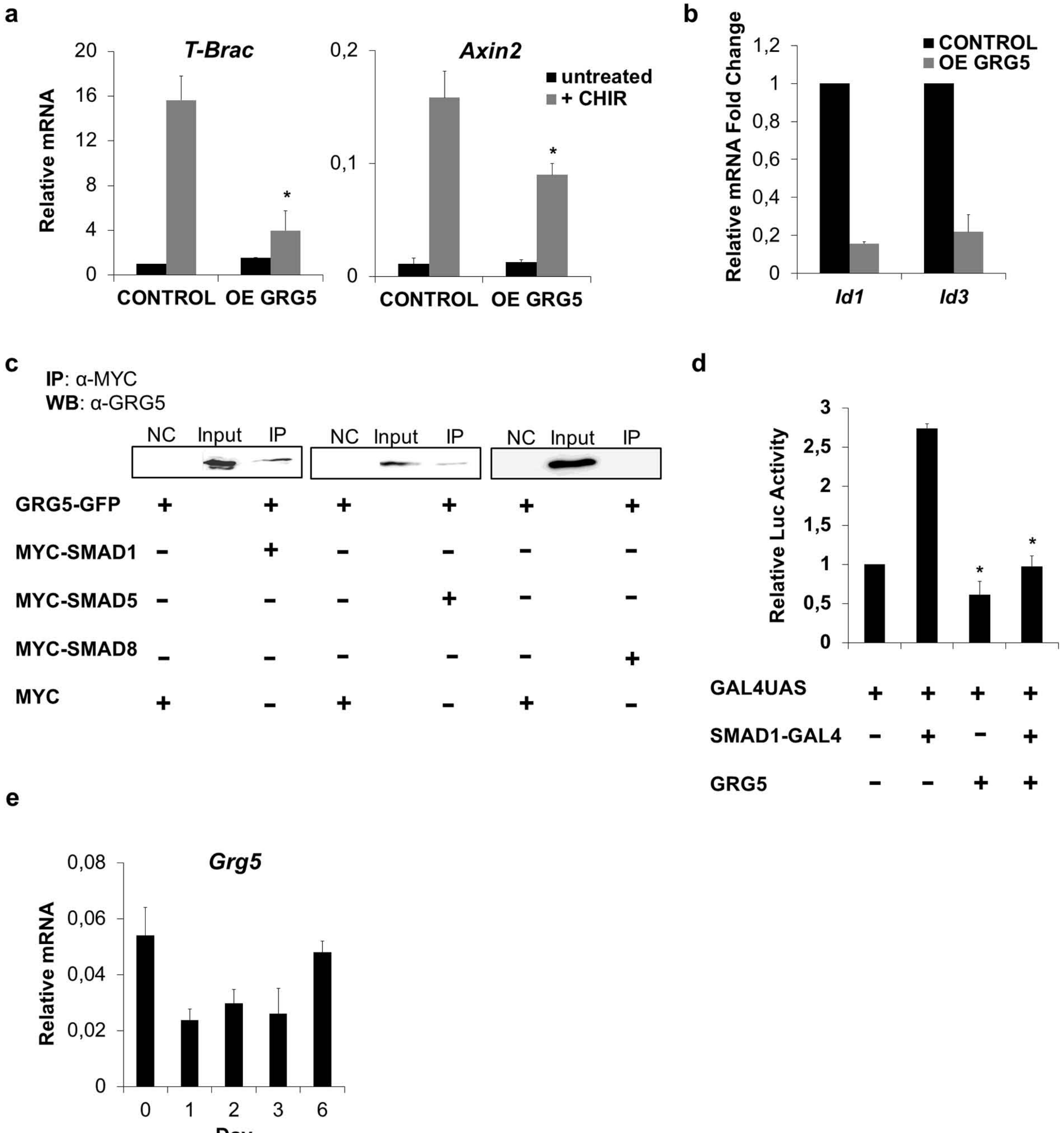

SI Figure 5: GRG5 hinders Bmp and Wnt signaling pathway activity thereby regulating ESC neuroectodermal commitment. (a) Relative mRNA levels of the Wnt pathway target genes *T-Brac* and *Axin2* in CONTROL and OE GRG5 ESCs upon signaling stimulation with 2μM CHIR. (b) Gene expression levels of the Bmp pathway targets *Id1* and *Id3* in CONTROL and OE GRG5 ESCs. (c) Co-immunoprecipitation assay showing interaction of GRG5 with SMAD1 and SMAD5 but not with SMAD8. Whole cell lysates of HEK293 cells, transfected with GRG5-GFP and MYC-SMAD1 or MYC-SMAD5, was immunoprecipitated with anti-MYC and immunoblotted with anti-GFP. HEK293 cells transfected with MYC and GRG5 GFP were used as negative control NC. (d) Lusiferase assay using the GAL4UAS system to show the transcriptional activity of SMAD1 upon transient overexpression of GRG5 in HEK293 cells. *P<0.05 (e) *Grg5* mRNA levels throughout ESC neural differentiation process. For a, b and e data are presented as mean + SD of n = 3 independent experiments.

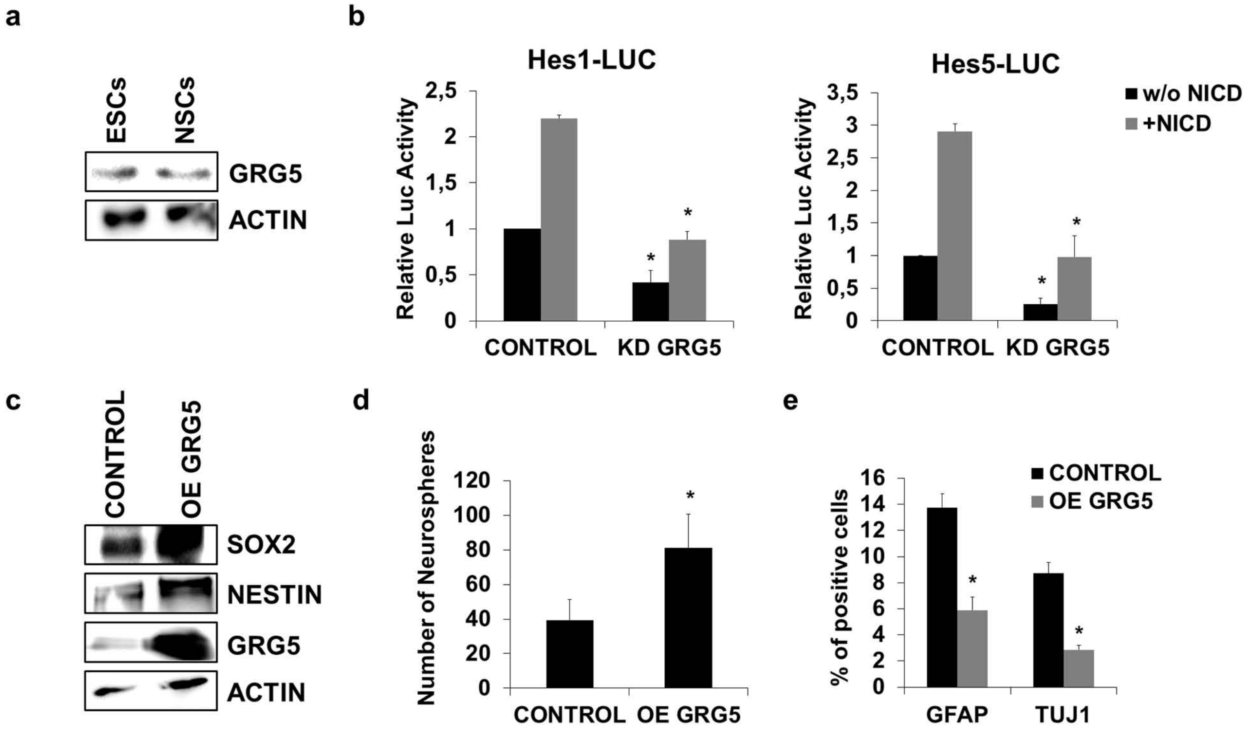

SI Figure 6: GRG5 promotes self-renewal of embryonic NSCs. (a) GRG5 protein levels in ESCs and NSCs. (b) Luciferase assay showing the activity of *Hes1* and *Hes5* promoters upon stimulation with NICD overexpression in control and KD GRG5 cells. mean + SD of n = 3 independent experiments. *P<0.05 (c) Western Blot showing expression of NSC factors in CONTROL and OE GRG5 NSCs. (d) Neurosphere forming assay comparing CONTROL and OE GRG5 NSC self-renewal capacity. Six days upon cell culture in proliferation medium, the total number of the formed neurospheres was counted. mean + SD of n = 3 independent experiments. *P<0.05 (e) OE GRG5 NSCs present impaired differentiation capacity. NSCs were differentiated for five days. Quantification of immunofluorescence staining for the astrocytic marker GFAP and the neuronal marker TUJ1 at Day5. mean + SD of n=3 independent experiments. *P<0.05

Supplementary Table S1: Antibodies

GRG5	IMG-5408, Imgenex
ACTIN	sc-47778, Santa Cruz
OCT4	sc-5279, Santa Cruz
NANOG	8600S, Cell Signaling
SOX2	27485, Cell Signaling
KLF4	D1F2, Cell signaling
c-MYC	D3N8F, Cell Signaling
p-STAT3 (Tyr705)	D3A7, Cell Signaling
p-STAT3 (Ser727)	9134, Cell signaling
CDH1	24E10, Santa Cruz
P21	C-19, Santa Cruz
ppRB	5807/811 Cell Signaling
pRB	D20, Cell Signaling
NrOB1	sc-13034X, Santa Cruz
NESTIN	MAB353, Millipore
GFAP	G3893, Sigma
NOTCH1	D1E11, Cell Signaling
CCND1	M-20, Santa Cruz
TUJ1	T8660, Sigma
MAP2	M2320, Sigma
SOX1	41945, Cell Signaling
CDX2	RM-2116-S, Thermo Scientific
DESMIN	M0760, DAKO
SYNAPTOPHYSIN	RM-9111-S, Thermo Scientific
Ki67	16A8, Biolegend

Supplementary Table S2: Primers

Wnt3a FOR	5’ AAA GTG TAA ATG CCA CGG GTT 3’
Wnt3a REV	5’ GGG ACT CAG GGT GTT TCT C 3’
Pax6 FOR	5’ GGTGCTGGACAATGAAAACA 3’
Pax6 REV	5’ GGTACAGACCCCCTCGGATAA 3’
Actinβ FOR	5’ GTGTGACGTTGACATCCGTA 3’
Actinβ REV	5’ GTAACAGTCCGCCTAGAAGC 3’
Id1 FOR	5’ GACTACATCAGGGACCTGCAGC 3’
Id1 REV	5’ GGCCGCCAAGGCACTGATCTCG 3’
Id3 FOR	5’ CCAGGTGGAAATCCTGCACC 3’
Id3 REV	5’ CTCTTGTCCTTGGAGATCACAA 3’
Sox17 FOR	5’ CTCTGCCCTGCCGGGATGG 3’
Sox17 REV	5’ AATGTCGGGGTAGTTGCAATA 3’
T-Brac FOR	5’ GTTCCCGGTGCTGAAGGTAAAT 3’
T-Brac REV	5’ GCGAGTCTGGGTGGATGTAGA 3’
ErbB3 FOR	5’ AAGGGTGTAAGGGACCAGAA 3’
ErbB3 REV	5’ AGTAGCGTCTCATAGCCCTT 3’
Talin FOR	5’ GCAGTCTCCGCTACCATAGA 3’
Talin REV	5’ ATGGCTCAAATTGCATCGTC 3’
Tcf7l1 FOR	5’ TCTGGATGAGGTCAAGTCGT 3’
Tcf7l1 REV	5’ GGGGCCATTTCATCTGTAGG 3’

Thbs1 FOR	5’ AGAGCATCTTCACCAGGGAT 3’
Thbs1 REV	5’ ATGTAGTTGGTGCGGATAGC 3’
Tcf7l2 FOR	5’ CATCCGCTAGGATGGTTAGT 3’
Tcf7l2 REV	5’ AGTCCTGATGCTTTGAGCTG 3’
Gata3 FOR	5’ GATGTAAGTCGAGGCCCAAG 3’
Gata3 REV	5’ ATTAGCGTTCCTCCTCCAGA 3’
Igf2 FOR	5’ GGAAAACGACTGGGCATTG 3’
Igf2 REV	5’ CCAAAGAGATGAGAAGCACCA 3’
Porcn FOR	5’ CTTCGCAAGTGGCTACGAG 3’
Porcn REV	5’ AGGCGAAGGGCATTTTTGA 3’
Smyd1 FOR	5’ GTGGACACGTTCTTGCAGTA 3’
Smyd1 REV	5’ CGGAGCTCAATCTTGCCATT 3’
Oct4 FOR	5’ CCCTGGGCGTTCTCTTTGGAA 3’
Oct4 REV	5’ ACCAGGGTCTCCGATTTGCAT 3’
Eomes FOR	5’ GCTTCCGGGACAACTACGA 3’
Eomes REV	5’ GAGAGGAGGCCGTTGGTCT 3’
Map2 FOR	5’ AGCCGCAACGCCAATGGATT 3’
Map2 REV	5’ TTTGTTCCGAGGCTGGCGAT 3’
Grg5 FOR	5’ AGAAGTCAGAGATGCAGAGGC 3’
Grg5 REV	5’ GGAGCTGCTGTCGGATGAT 3’
Bmp4 FOR	5’ TTCCTGGTAACCGAATGCT 3’
Bmp4 REV	5’ AAGTGTCGCCTCGAAGTC 3’
Sox2 FOR	5’ AGCTCGCAGACCTACATGA 3’
Sox2 REV	5’ TGGCCTCGCACTTGAC 3’
Tuj1 FOR	5’ AAGGTAGCCGTGTGTGACATC 3’
Tuj1 REV	5’ ACCAGGTCATTCATGTTGCTC 3’
Ngn2 FOR	5’ GCTGGCATCTGCTCTATTCC 3’
Ngn2 REV	5’ ATGAAGCAATCCTCCCTCCT 3’
Flk1 FOR	5’ GGATGGAGGCCTCTACACC 3’
Flk1 REV	5’ TGCCGACGAGGATAATGAC 3’
Gsc FOR	5’ TGCTGCCCTACATGAACGTG 3’
Gsc REV	5’ CTCCAGGGCTTCGAGCTG 3’
Nrob1 FOR	5’ CTGGTGTGCAGCGTCTGA 3’
Nrob1 REV	5’ GTGTTGGTCTCCGGGATCTC 3’
Klf4 FOR	5' AGAGGAGCCCAAGCCAAAG 3'
Klf4 REV	5' GGTTTCTCGCCTGTGTGAGTT 3'
Gata 4 FOR	5’ GCCAACTGCCAACTACCAC 3’
Gata4 REV	5’ GACCTGCTGGCGTCTTAGA 3’
